# Untargeted Metabolomics Unveil Changes in Autotrophic and Mixotrophic *Galdieria sulphuraria* Exposed to High-Light Intensity

**DOI:** 10.3390/ijms22031247

**Published:** 2021-01-27

**Authors:** Lu Liu, Carlos Sanchez-Arcos, Georg Pohnert, Dong Wei

**Affiliations:** 1 School of Food Science and Engineering, South China University of Technology, Wushan Rd. 381, Guangzhou 510641, China; liululucia@scut.edu.cn; 2 Guangdong Provincial Key Laboratory of Microbial Culture Collection and Application, State Key Laboratory of Applied Microbiology Southern China, Guangdong Institute of Microbiology, Guangdong Academy of Sciences, Guangzhou 510070, China; 3 Aquatic Chemical Ecology, Cologne Biocenter, University of Cologne, 50674 Cologne, Germany; c.sanchezarcos@uni-koeln.de; 4 Institute for Inorganic and Analytical Chemistry, Bioorganic Analytics, Friedrich Schiller University Jena, Lessingstr. 8, 07743 Jena, Germany; georg.pohnert@uni-jena.de; 5 Research Institute for Food Nutrition and Human Health, Guangzhou 510640, China

**Keywords:** high-light, growth, extremophile, metabolomics, *Galdieria sulphuraria*

## Abstract

The thermoacidophilic red alga *Galdieria sulphuraria* has been optimizing a photosynthetic system for low-light conditions over billions of years, thriving in hot and acidic endolithic habitats. The growth of *G. sulphuraria* in the laboratory is very much dependent on light and substrate supply. Here, higher cell densities in *G. sulphuraria* under high-light conditions were obtained, although reductions in photosynthetic pigments were observed, which indicated this alga might be able to relieve the effects caused by photoinhibition. We further describe an extensive untargeted metabolomics study to reveal metabolic changes in autotrophic and mixotrophic *G. sulphuraria* grown under high and low light intensities. The up-modulation of bilayer lipids, that help generate better-ordered lipid domains (e.g., ergosterol) and keep optimal membrane thickness and fluidity, were observed under high-light exposure. Moreover, high-light conditions induced changes in amino acids, amines, and amide metabolism. Compared with the autotrophic algae, higher accumulations of osmoprotectant sugars and sugar alcohols were recorded in the mixotrophic *G. sulphuraria*. This response can be interpreted as a measure to cope with stress due to the high concentration of organic carbon sources. Our results indicate how *G. sulphuraria* can modulate its metabolome to maintain energetic balance and minimize harmful effects under changing environments.

## 1. Introduction

Specialized microorganisms that populate harsh environments characterized by extreme temperature, pH, and salinity, are called extremophiles [1]. Although most of them are prokaryotic, some eukaryotes also belong to this group of microorganisms. *Galdieria sulphuraria* is a eukaryotic red alga belonging to the Cyanidiales family. As an extremophile, it is adapted to scorching temperatures (up to 56 °C) and acidic pH (0.5–3.0) [2]. This alga is the only species of the Cyanidiales that can grow autotrophically, mixotrophically, and heterotrophically. It possesses the ability to tolerate various stresses and accumulate beneficial compounds [3]. *G. sulphuraria* is considered a new model organism for biotechnology due to its tolerance mechanisms to environmental stresses [4]. However, the mechanisms of adaptation are currently poorly understood. Further investigations are required before full biotechnological potential can be reached.

To survive under biotic and abiotic stress conditions, microorganisms have developed adaptation mechanisms by modifying gene expression, synthesizing proteins, and ultimately modulating levels of specific metabolites in crucial pathways [2,5]. Extremophiles have evolved various metabolic adaptation mechanisms that allow them to thrive in extreme habitats facing unfavorable growth conditions. Recently, metabolomics has provided new insight into identifying metabolic responses to stress in extremophiles. For instance, the metabolomic analysis suggested that levels of di-myo-inositol phosphate, mannosylglycerate, UDP-N-acetylglucosamine, and UDP-N-acetylgalactosamine were enhanced considerably in *Pyrococcus furiosus* in response to heat stress [6]. Empirical evidence suggests that metabolites including proline, proline analog, glycine betaine, pinitol, myo-inositol, mannitol, sorbitol, O-methylmucoinositol, and polyamines are essential for halophytes to grow in high saline conditions [7]. Furthermore, metabolomics analyses were used to support the elucidation of survival strategies modulated by an intracellular compound in snow algae, a group of poly-extremophilic organisms living in low temperatures, high irradiation, and low pH snowfields and glacier surfaces [8,9].

The dynamic growth of microalgae is very much dependent on light and substrate supply. *G. sulphuraria* thrives in a natural endolithic habitat that is extremely hot and acidic and under consistent low-light intensity. It has been reported in the literature that the growth of this algae is strongly inhibited under high-light intensity because they lack an LHCII gene, which is pivotal for the ability to adapt to changing light conditions [10]. Conversely, in our lab, high cell densities of another *G. sulphuraria* strain were obtained in autotrophic and mixotrophic conditions under high-light stress, which indicated this alga might be able to relieve the effects caused by photoinhibition. Metabolomic analyses could provide a comprehensive insight into understanding dynamic networks of metabolites in *G. sulphuraria* induced by high-light. In the current study, we performed a liquid chromatography–mass spectrometry (LC–MS) and gas chromatography–mass spectrometry (GC–MS)-based untargeted metabolomic approaches to identify metabolic changes in the endometabolome of autotrophic and mixotrophic *G. sulphuraria* by high-light intensity.

## 2. Results

### 2.1. Physiological Status

**Figure 1:**
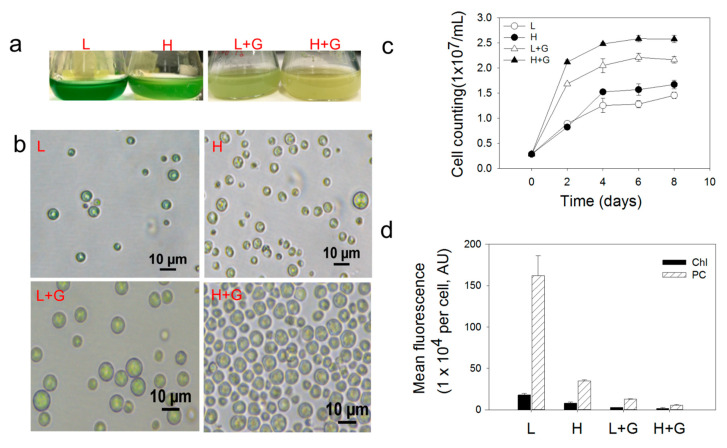
Images of *G. sulphuraria* liquid cultures (**a**), microscopic cell images (**b**), cellular growth curves (**c**), and bar plot of the mean fluorescence intensities of chlorophyll (Chl) and phycobilin (PC) photosynthetic pigments (**d**), under low-light intensity (L), low-light intensity with glucose addition (L + G), high-light intensity (H), high-light intensity with glucose addition (H + G). Error bars indicate the standard deviation (*n* = 6).

Over the whole process of cultivation, we observed significantly faster growth and larger cell sizes in mixotrophic cultures than autotrophic cultures. The high-light intensity led to growth increases of mixotrophic and autotrophic cultures compared to low-light intensity conditions (Figure 1c). Mean intracellular microalgal photosynthetic pigments were significantly higher in the autotrophic cultures compared to cells grown under mixotrophic conditions and were always lower under high-light intensity in comparison to low-light intensity conditions (Figure 1d). Algal cells turned from green-blue to pale yellow color by applying high-light intensity and the addition of glucose (Figure 1a,b). In autotrophic cultures, a decrease of 55.4% in chlorophyll (Chl) fluorescence and 78.4% in phycobilin (PC) was observed under high-light intensity compared to low-light intensity. The photosynthetic pigments decline even more when comparing cultures with and without glucose. Mean Chl and PC fluorescence in low-light mixotrophic cultures showed drops of 82.9 and 92.2%, respectively, compared to autotrophic cultures (Figure 1d).

### 2.2. GC–MS Endo-Metabolic Profiles

**Figure 2:**
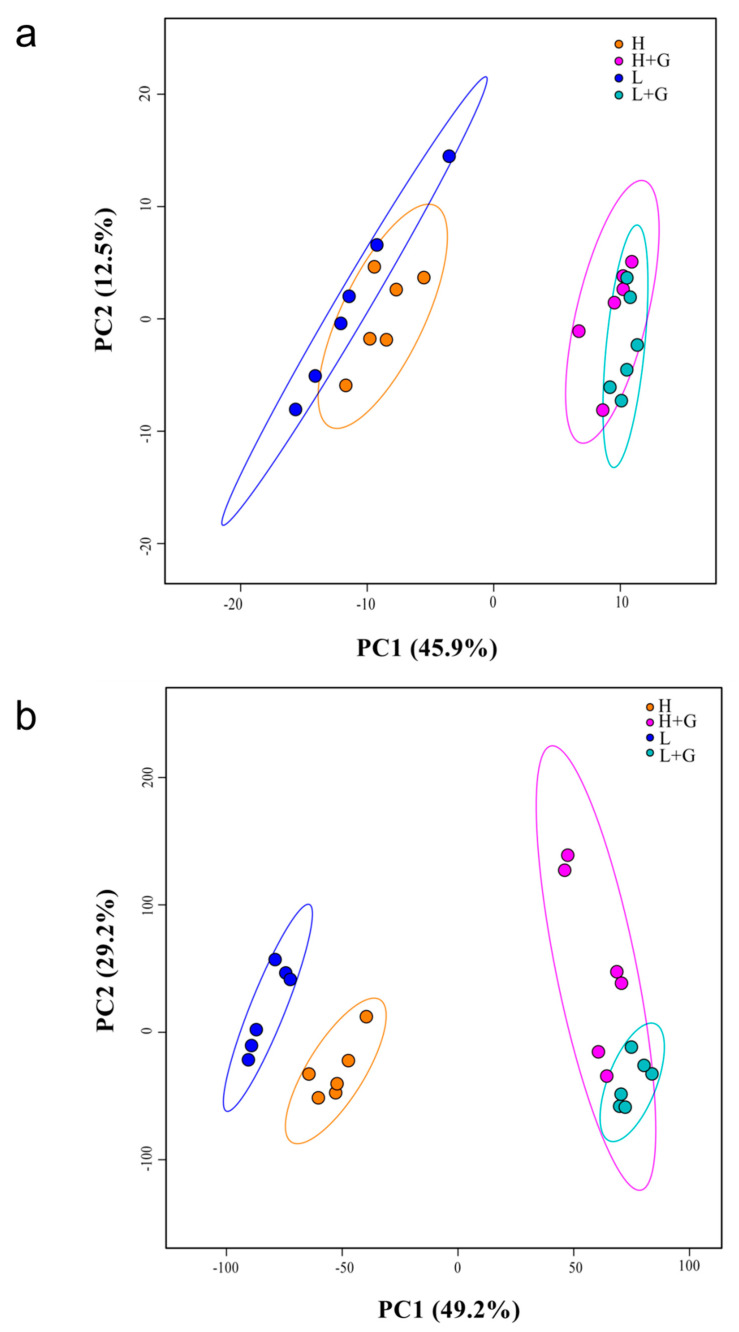
Principal component analysis (PCA) score plots of intracellular metabolomic profiles of *G. sulphuraria* under low-light intensity (L), low-light intensity with glucose addition (L + G), high-light intensity (H), high-light intensity with glucose addition (H + G) culture conditions. Metabolomics data were collected by (**a**) GC–MS and (**b**) LC–MS.

Metabolic profiling of intracellular extracts of *G. sulphuraria* cells from our four treatments using GC–MS led to a total of 237 peaks after data analysis. A principal component analysis (PCA) was carried out to determine the relationships among the non-polar metabolic profiles of *G. sulphuraria* under all treatments. The principal component 1 (PC1), from the PCA plot (Figure 2a), explains 45.9% of the variability of the total data and separates not only the metabolic profiles of *G. sulphuraria* without glucose supplementation (H and L) in the left from those with glucose supplementation (H + G and L + G) in the right, but also, to a lesser extent, splits up the treatments under low-light (L) from high-light (H). The separations observed in PC1 suggest that the intracellular metabolome of *G. sulphuraria* significantly changes by supplementing the cultures with an external carbon source and by exposing the cultures to a higher intensity of light (Figure 2a). To further verify the observed separations between two different treatments, PCAs for two-group comparisons were carried out. As shown in Figure S1a, the PC2 explained 25% of the total variation and revealed two distinct groups associated with the high-light (H) and low-light (L) treatments. In Figure S1b, PC1 explains 55.8% of the total data variability and separates the metabolic profiles of *G. sulphuraria* with glucose (right) from those without glucose (left) under low-light intensity conditions. The PC1 in Figure S1c explains 43.9% of the variability across the data set, separating the metabolic profiles of autotrophic (left) and mixotrophic (right) *G. sulphuraria* exposed to high-light. Although the metabolic profiles of mixotrophic *G. sulphuraria* under high-light and low-light intensity conditions did not separate in a manner as pronounced as in the other comparisons, the two treatments clustered apart (Figure S1d).

To confirm the observed differences, a partial least squares-discriminant analysis (PLS-DA) and a *t*-test were performed. Metabolites with VIP > 1 and *p* < 0.05 were selected as differentially modulated from our four comparisons. Tables S1–S4 contain the putative identification of selected differentially modulated metabolites. Comparing between autotrophic *G. sulphuraria* at high-light and low-light intensities, 35 compounds were differentially modulated, 26 from those metabolites were putatively identified using library data. Twenty of the putatively identified metabolites were up modulated, and six metabolites down modulated. When mixotrophic and autotrophic *G. sulphuraria* under low-light intensity conditions were compared, 107 metabolites were found differentially modulated, among which 61 were putatively identified (Table S2). For the same cultures under high-light intensity conditions, 89 metabolites were significantly modulated, of which 47 were putatively identified. Correlating with the reduced separation observed in the PCA plot, only eight compounds were found as differentially modulated in response to changing light intensity for mixotrophic *G. sulphuraria*. From all putatively identified compounds, the chemical identities of 16 metabolites were confirmed based on authentic standards injected under the same analytical parameters (Supplementary Table S5). For instance, the Trimethylsilyl (TMS) derivative of ergosterol, one of the major significantly modulated compounds, was confirmed by matching mass spectra, a retention time of 38.57 min, and a Kovats index of 3232.

**Figure 3:**
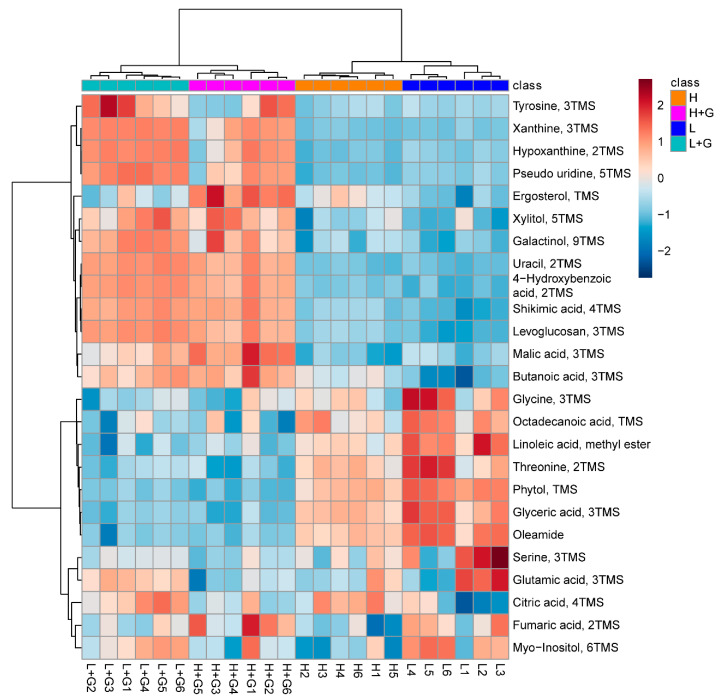
Heat map with the top 25 most significantly modulated metabolites in *G. sulphuraria* (GC–MS data) according to the partial least square discriminant analysis variable importance in projection scores under low-light intensity (L), low-light intensity with glucose addition (L + G), high-light intensity (H), high-light intensity with glucose addition (H + G) culture conditions. Structures were tentatively assigned according to library data.

The heatmap in Figure 3 graphically depicts the abundances of the top 25 most discriminating metabolites (putatively identified using library data) among the four treatments. The metabolic profiles were grouped into two main clusters. A first cluster consisted of the mixotrophic cultures and a second cluster of the autotrophic ones. The most discriminant compounds were divided into three regions of interest. Starting from the top, the first region contains, among others, organic acids (e.g., malic acid, butanoic acid, shikimic acid, and hydroxybenzoic acid) and purines (e.g., xanthine, hypoxanthine), present at higher levels when *G. sulphuraria* grows in mixotrophic cultures than under autotrophic conditions. Ergosterol, the precursor of vitamin D2, displayed elevated levels when algae are exposed to high-light. Ergosterol levels increased substantially when glucose was added to the media (mixotrophic culture). In the second region of metabolites, amino acids (glycine and threonine), fatty acids (octadecanoic acid and linoleic acid), glyceric acid, phytol, and oleamide, were detected in reduced quantities when *G. sulphuraria* was grown under mixotrophic conditions in comparison with autotrophic cultures.

### 2.3. LC–MS Endo-Metabolic Profiles

LC–MS-based untargeted metabolomics was used to reveal differences between the polar and semipolar metabolites from *G. sulphuraria*. Data analysis of the metabolic profiles from our four treatments delivered 245 and 193 features in positive and negative mode, respectively. PCA scores plot (Figure 2b) for the metabolic profiles of *G. sulphuraria* growing under different conditions showed that the PC1 accounted for 49.2% of the total data variance, again separating the metabolic profiles of cultures without glucose at the left from those with glucose on the right. As for the GC–MS data, the algal metabolic profiles growing under low-light and high-light intensities were also grouped apart. The verification of the observed separations by comparing sets of two different treatments is shown in the supplementary Figure S2. In the PCA shown in Figure S2a, the PC1 accounted for 56.5% of the total variation. It revealed two distinct groups associated with algae grown under low-light (right) and high-light (left) intensity conditions. In Figure S2b, the metabolic profiles of autotrophic *G. sulphuraria* cultures (left) were separated from those of mixotrophic cultures (right), both under low-light intensity conditions by the PC1 (71.5%). The PC1 shown in Figure S2c explains 67.6% of the variation across the data set under high glucose and high vs. low light intensity conditions. Similar to what was observed for the GC–MS data, the LC–MS metabolic profiles of mixotrophic *G. sulphuraria* under high-light and low-light intensity conditions did not separate as pronouncedly as in the previous comparisons. Still, the two treatments were also clustered apart (Figure S2d).

Table 1 lists the most differentially modulated metabolites for the comparisons mentioned above. Under autotrophic *G. sulphuraria* cultures and high-light intensity exposure, Lysophosphatidylcholine (LysoPC) (18:3), LysoPC (18:2), LysoPC (18:1), and PC (18:1/20:6) were up modulated with fold changes of log2 (Fold Change) >2. Three compounds were down modulated, one of which was putatively identified as phosphoinositol (22:6/22:4) compared to those cultures under low-light intensity. Compared to L group samples, L + G treatment significantly up modulated two phospholipids, LysoPC (18:3) and PC (18:2/20:6). In contrast, two peptides, a porphyrin derivative, a glycerophosphoinositols, and a lysophosphatidylethanolamine, were significantly down modulated under the same conditions. The supply of glucose under high-light intensity conditions up modulated only LysoPC (18:3). All the other modulated compounds displayed significantly lower levels after adding glucose to the cultures than autotrophic conditions (H). Finally, when comparing the mixotrophic cultures at low-light and high-light intensities, a set of phospholipids, including LysoPC, PC, Lysophosphatidylethanolamine (LysoPE), and phosphoinositol, were down modulated by increasing the intensity of the light.

### 2.4. Metabolic Pathway Analysis

**Figure 4:**
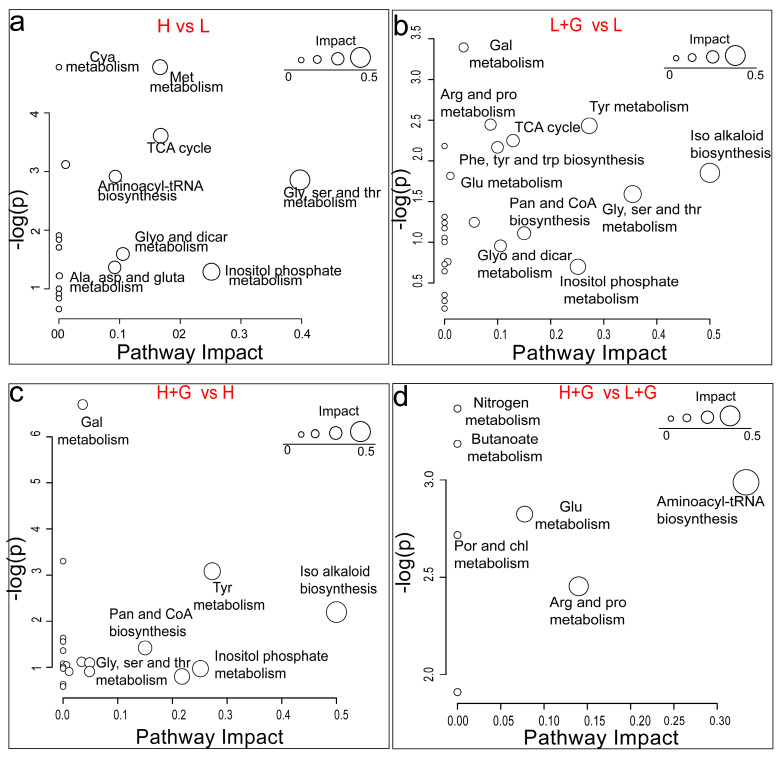
Overview of the most differentially modulated pathways of *G. sulphuraria* cultures under the different culture conditions based on GC–MS data. Low light intensity (L), low-light intensity with glucose addition (L + G), high-light intensity (H), high-light intensity with glucose addition (H + G) conditions. Cyanoamino acid (Cya); methane (Met); glutathione (Glu); glycine (Gly); serine (Ser); threonine (Thr); glyoxylate (Glyo); dicarboxylate (Dicar); alanine (Ala); aspartate (Asp); glutamate (Glu); galactose (Gal); arginine (Arg); proline (Pro); pyrimidine (Pyr); tyrosine (Tyr); phenylalanine (Phe); tryptophan (Trp); pantothenate (Pan); porphyrin (Por); chlorophyll (Chl); isoquinoline (Iso). Differential pathways between: (**a**): H vs L; (**b**): L + G vs L; (**c**): H + G vs H; (**d**): H + G vs L + G.

To bring our metabolomic results into a biological context, we investigated which metabolic pathways are significantly impacted in *G. sulphuraria* by the different illumination intensities and the addition of an external carbon source (Figure 4). Only differentially modulated metabolites (GC data) putatively identified above level two were selected for the pathway analysis. Our study recorded decreased levels of glycine and serine under high-light exposure. These two amino acids are linked to several pathways, such as cyanoamino acid metabolism and aminoacyl-tRNA biosynthesis (Table S6). Similarly, inositol phosphate metabolism is impacted by high-light intensity related to myoinositol’s down-modulation. Associated with the down-modulation of oxoglutaric acid and up modulation of citric acid, the tricarboxylic acid (TCA cycle) was found to be affected (Figure 4a).

The addition of an external carbon source as glucose at low-light intensity conditions significantly impacted the isoquinoline alkaloid biosynthesis in *G. sulphuraria*, associated with the up-modulation of tyrosine. Glycine, serine, and threonine metabolism were also impacted considerably but linked to glycine and L-threonine down-modulation. Mixotrophic conditions with low light also increased the level of myoinositol involved in the pathway of inositol phosphate metabolism. This experimental condition also impacted the TCA cycle by up-modulating citric acid and fumaric acid. The latter compound was also involved in tyrosine metabolism (Figure 4b). The same metabolic pathways were the most significantly impacted when adding glucose at high-light intensity conditions and linked to the same tendencies and compound modulations (Figure 4c). Finally, the increase in the light intensity in mixotrophic cultures of *G. sulphuraria* significantly impacted the aminoacyl-tRNA biosynthesis, arginine and proline metabolism, glutathione metabolism, and porphyrin and chlorophyll metabolism, linked all to the down-modulation in the levels of glutamic acid (Figure 4d).

## 3. Discussion

### 3.1. G. sulphuraria Modulates Membrane Lipid Composition to Cope with High-Light Intensity Stress

**Figure 5:**
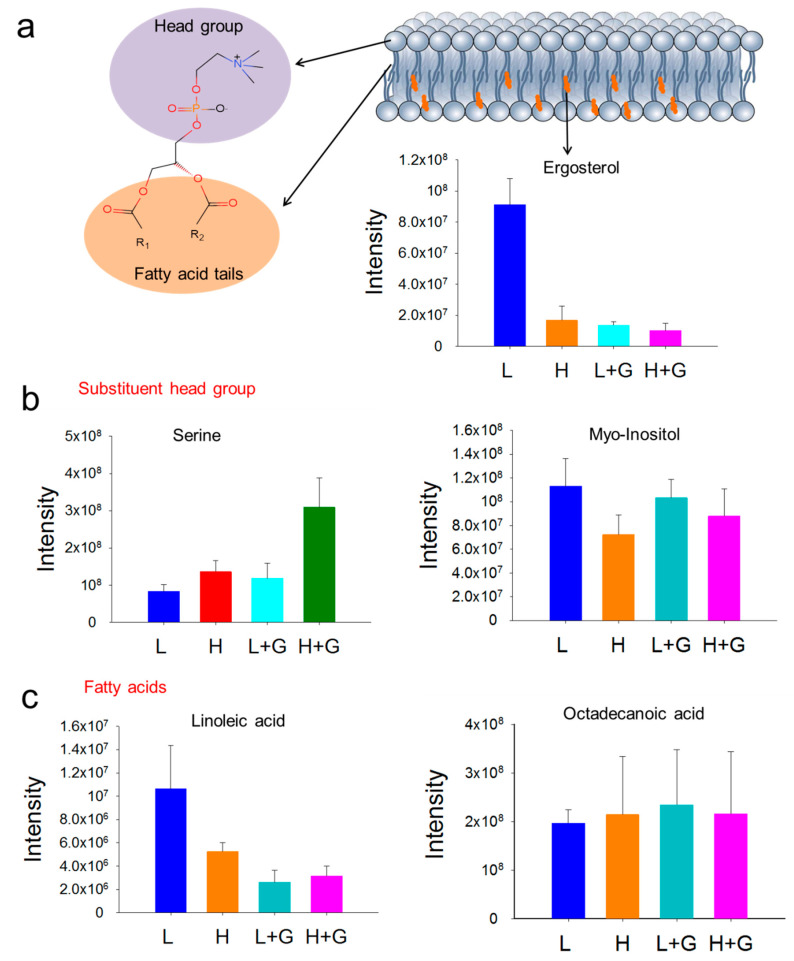
Remodeling of *G. sulphuraria* membrane lipids. The basic structure of glycerolphospholipids from lipid bilayer membranes and bar plot (GC-MS data) of ergosterol abundances (**a**); Bar plot of the abundances of various head group metabolites (**b**) and bar plots of fatty acids tails abundances (**c**), under low-light intensity (L), low-light intensity with glucose addition (L + G), high-light intensity (H), high-light intensity with glucose addition (H + G) culture conditions.

Light supply is an essential factor affecting algal growth and its metabolome [11]. Over billions of years, *G. sulphuraria* has thrived in the stable low-light endolithic habitats provided by volcanic rock crevices, while in lab cultivation, their growth showed several possible types [10]. It has been reported in the literature, that *G. sulphuraria* can grow under photoautotrophic, heterotrophic, and mixotrophic conditions and balance light-harvesting and energy utilization [4]. Remodeling membrane organization is crucial for the survival of microalgae when exposed to habitat changes such as light, temperature, and nutrient availability [12]. Our results indicate that changes in membrane composition might provide an important survival factor for *G. sulphuraria* to deter the stress caused by high-light exposure. Like most secondary bioactive metabolites in algae, sterol up-regulation is mainly attributed to the extreme and hostile environment [13]. These compounds are a vital component of cell membranes and responsible for various cell properties and functions [14]. Algae contain diverse types of sterols, which are usually relatively stable; however, they can be influenced by numerous biotic and abiotic factors [15]. Green algae, such as *Dunaliella tertiolecta*, contain ergosterol as one of the predominant sterols in the membrane, while generally, cholesterol was the primary compound in red algae species [16,17]. As one of the significant membrane lipids, ergosterol, a precursor of vitamin D2, is mainly produced by fungi but has also been found in plants and algae associated with other phytosterols [18]. In comparison with cholesterol, ergosterol displays a higher ordering effect on the phospholipid acyl chains, generating relatively better-ordered lipid domains (also known as lipid rafts) [19]. Our study demonstrates that high-light intensity affects the levels of membrane lipids in *G. sulphuraria*, mainly ergosterol and phospholipids (Figure 5). Moreover, we observed significantly up-modulated levels of bilayer-preferring lipids, such as *glycerophosphocholines* (PC) and glycerophosphoinositols (PI), which have an essential function to form a stable matrix for cellular membranes [20].

Moreover, in our study, increased lysophosphatidylcholine levels (LysoPC, a monoacylated form of PC) were observed when *G. sulphuraria* cultures were exposed to high-light intensity or high levels of glucose. It has been proposed that lysoPCs act as signal compounds in plant stress response mechanisms [21]. Lysophospholipids, intermediates of phospholipid remodeling, are regulated by degradation and acylation, related to the activities of phospholipases A, acyltransferases, and lysophospholipases [22]. *Parachlorella kessleri* reorganized membrane lipid under salt stress by up-regulated genes encoding phospholipase A2 and lysophospholipid acyltransferases [23]. Significant differences were found in the polar lipids composition of *Saccharina latissima* originating from three distinct locations (France, Norway, and the United Kingdom). Samples from northwestern France, with higher light intensity and temperature amplitude, were enriched in lyso lipids compared to samples of other origins [24]. In summary, our results indicate that *G. sulphuraria* can remodel the membrane lipid constitution when exposed to high-light intensities to maintain optimal membrane properties.

### 3.2. Light Stress Induces Changes in Amino Acid, Amine, and Amide Metabolism

Rapid protein degradation and replacement have been well documented as a photoprotection strategy in photosynthetic plants, algae, and cyanobacteria as a protection strategy of photoinhibition [25]. The decrease in the abundance of phycobiliproteins, serving as primary light-harvesting antennae for photosystem II, could result from photoprotection in response to damage caused by high-light exposure [26]. In our study, *G. sulphuraria* exhibited a modulation of amino acid pools as one of the most significant metabolic changes in response to high-light exposure. Under high-light stress, cells might have a higher growth rate and a bulk demand for metabolic activity and energy supply [27]. To meet increased respiration, glycine was significantly reduced under high-light conditions, in concert with the decrease in serine, likely reflecting an increase in the demand to form pyruvate, which is the principal substrate of the TCA cycle [28]. There are also transient increases in aspartate-derived amino acids such as leucine, possibly due to the hormonal balance regulation to minimize the damage because of high-light exposure [29].

Polyamines (PAs), similarly to hormones, regulate a wide array of fundamental processes in plants such as cell division, differentiation, membrane stabilization, reproduction, senescence, and homeostatic adjustments in response to abiotic and biotic stresses [30]. They are also involved in chlorophyll biosynthesis and the assembly of photosynthetic membrane complexes [31]. In our study, a higher relative amount of putrescine was observed in high-light exposed *G. sulphuraria*. As a well-known PA, it has shown a substantial role in protecting plants’ photosynthetic apparatus under stress conditions such as UV-B radiation and ozone pollution [32,33].

Moreover, the relative abundance of niacinamide (NIC) was also recorded at increased levels under high-light. NIC plays an essential role in the induction of defensive metabolism in eukaryotic cells. In *Pisum sativum*, various levels of mRNAs for defense-involved genes were up-regulated by NIC [34]. Similarly, we also observed an up modulation of oleamide levels. These changes in amino acid, amine, and amide metabolism might result from high-light intensity exposure as an effort to balance energy utilization and enhance tolerance to prevent oxidative stress.

### 3.3. Mixotrophic Environments Promote Alterations in Energy Metabolism

Mixotrophy, with CO_2_ and exogenous organic carbon as carbon sources, combines autotrophic and heterotrophic modes of nutrition [35]. *G. sulphuraria* is the only member of the Cyanidiales capable of a mixotrophic lifestyle, using a range of organic carbon sources, including glucose under light supplementation [36]. However, excess glucose and light supply inhibited the photopigments’ biosynthesis, attributed to the inactivation of the oxygen-dependent coproporphyrinogen III oxidase. This enzyme converts coproporphyrinogen III to protoporphyrinogen IX, a critical step in chlorophyll and phycobilin biosynthesis [37]. Thus, the observed decrease in photopigments chlorophyll and phycobilin is most likely due to the limited oxygen for the oxygen-dependent oxidoreductase.

To sustain viability during hypoxia/anoxia, most of the oxygen might be used by the microalgae to support glucose conversion into energy by glycolysis, pyruvate metabolism, and the Krebs cycle for the production of ATP and recycle the NAD(P)H and FADH2 [38]. In our study, excess of glucose and the lack of oxygen might also lead mixotrophic *G. sulphuraria* to not utilize a complete TCA cycle, which could explain the significantly increased levels of intermediates involved in the TCA cycle pathways. Moreover, mixotrophic conditions also impacted purine metabolism with xanthine and hypoxanthine modulations. These two purine bases occur as intermediates involved in nucleic acid formation through the salvage pathway, an essential energy-saving route of nucleotide biosynthesis in plant cells [39].

In plants, the shikimate pathway is closely interlinked with the biosynthesis of aromatic amino acids, which serve as precursors for secondary metabolites with diverse physiological roles providing ultraviolet protection, electron transport, and signaling molecules [40]. In the present study, we found a significant up-modulation in the abundance of shikimate, an essential compound in the shikimate pathway converted from 3-dehydroshikimic acid (DHS). The increased accumulation of the downstream product aromatic amino acid L-tyrosine and aromatic intermediate 4-aminobenzoic acid were also recorded in mixotrophic *G. sulphuraria*. Under normal conditions, the total fixed carbon flow through the shikimate pathway counts as 20%. Higher carbon flow through the pathway is thought to occur under times of plant stress or rapid growth [41]. Therefore, the observed increase in intermediates in the shikimate pathway is likely due to the bulk of carbon flow to meet the rapid growth observed under our high-light intensity and glucose supplementation treatments.

To maintain cellular homeostasis under stress conditions, plants have developed several strategies, such as sugar metabolism regulation to adjust osmotic pressure. In our study, *G. sulphuraria* at mixotrophic conditions showed an accumulation of sugars, such as tentatively assigned levoglucosan, allopyranose, and gentiobiose, with the latter known as an osmoprotectant and stress marker [42]. Additionally, some sugar alcohols, putatively identified as sorbitol, glucitol, xylitol, galactinol, and threitol, increased in algae at mixotrophic cultures. In general, sugar alcohols exhibit multiple functions in plants and algal metabolism. They have been demonstrated to play essential roles in various stress responses, especially in osmotic stress [43]. For example, it is reported that galactinol can act as a protectant for plants from oxidative damage [44]. In our study, the increase in sugar alcohols in algae under mixotrophic conditions might be associated with osmotic stress due to the high concentration of organic carbon sources. The presence of polyols was suggested to remove the excess reducing equivalents and keep the relatively stable redox status in algae [27]. Our results suggest a strong network between carbohydrate metabolic pathways, such as the TCA cycle and sugar metabolism, and osmotic stress tolerance in *G. sulphuraria* to minimize the harmful effects of high-light intensity and an excess of carbon source.

## 4. Materials and Methods

### 4.1. Algal Culture and Growth Conditions

For this study, *Galdieria sulphuraria* UTEX 2919 was obtained from UTEX Culture Collection of Algae at the University of Texas. Cells were grown for seed culture in 2 × Allen’s Medium [36] in 50 mL Schott Duran glass flasks (Mitterteich, Bayern, Germany) and incubated in a shaking incubator (Edmund Bühler GmbH KS-15, Bodelshausen, Germany) at 150 r/min and 40 °C under continuous light (15–25 μEm^−2^s^−1^) provided by a Philips cool white lamp MASTER TL Mini Super 80 8W/840 1FM/10X25CC (Burgebrach, Bavaria, Germany). For the mixotrophic culture, glucose (10 gL^−1^) was used as a carbon source. To evaluate the metabolic differences under light intensity stress, four different treatments were set as following: L: autotrophic culture under low light intensity (15–25 μEm^−2^s^−1^); L + G: mixotrophic culture under low light intensity (15–25 μEm^−2^s^−1^); H: autotrophic culture under high-light intensity (65–85 μEm^−2^s^−1^); H + G: mixotrophic culture under high-light intensity (65–85 μEm^−2^s^−1^). Cultures were inoculated with an initial cell concentration of about 2.87 × 10^6^ cells /mL. Six replicates were carried out for each treatment.

### 4.2. Physiological Characterization

To measure cell growth, algal cells were counted using a Fuchs-Rosenthal hemocytometer. To obtain a record of the cells’ physiological state, images were captured on a microscope of Leica microsystems CMS GmbH (Mannheim, Baden-Württemberg, Germany). To measure the relative quantity of photosynthetic pigments, BD Accuri C6 Plus flow cytometry (San Jose, CA, USA) was used according to the manufacturer’s recommendations. Emission levels of chlorophyll (Chl) and phycobilins (PC) were detected in channel FL3 (488 nm excitation, 670 LP filter) and FL4 (640 nm excitation, 675 ± 12.5 filter), respectively.

### 4.3. Algae Harvesting and Sample Preparation

After four days cultivation under the previously described treatments, algal cells were collected and extracted for metabolomic analysis. For LC–MS analysis, 3 × 10^8^ cells were collected per biological replicate, while 1.5 × 10^7^ cells per sample were sufficient for GC–MS analysis. Cells were harvested via filtration using Whatman GF/F filters (Darmstadt, Hessen, Germany) under reduced pressure (600 mbar). Afterward, the filters with algae cells were washed with 200 mL Milli-Q water and carefully transferred with tweezers to pre-chilled 25 mL glass beakers containing 1 mL of freshly prepared extraction mix (80% aqueous methanol for LC–MS and a solution of methanol: ethanol: chloroform in a 2:6:2 ratio, for GC–MS). Cells were resuspended by pipetting and incubated on ice for 15 min. The mixture was then transferred to 1.5 mL Eppendorf centrifuge tubes. As a negative control, 5 mL of 2 × Allen’s Medium was filtered and treated with solvents as described for the samples.

The cells’ lysis and extraction were carried out by adding 100 mg 0.5 mm diameterglass beads (Bartlesville, OK, USA) to each tube and shaking in a Qiagen TissueLyserII (Hilden, North Rhine-Westphalie, Germany) with a pre-cooled tube holder for 15 min at 30 Hz. All ground cell material was then separated by centrifugation (30,000× *g*, 4 °C, 15 min) at 4 °C for 10 min. Supernatants were transferred into 1.5 mL glass vials. According to cell numbers, QC samples were prepared from pooled samples by collecting equal aliquots from all samples. The LC samples were directly evaporated to complete dryness in a vacuum concentrator. In contrast, for GC samples, 5 µL of a ribitol 40 µM solution was added as an internal standard before the drying process. All the samples were filled with argon before taking out from the vacuum concentrator. Samples were stored at −20 °C for both platforms for further analytical analysis.

### 4.4. GC–MS Data Acquisition

For initial derivatization, 20 µL of the reagent methoxyamine hydrochloride in pyridine (20 mg mL^−1^) were added to each dry sample, first incubated at 60 °C for 1 h, and later kept at room temperature overnight. A blank sample was prepared by adding only derivatization reagents. For the Kovats index determination, one of the QC samples was spiked with 1 µL RI (retention index) mix (C7–C40, 100 µg mL^−1^ in hexane, Sigma-Aldrich, St. Louis, MO, USA). For the second derivatization step, automatic online derivatization was carried out with a TriPlus RSH™ Autosampler (Thermo Fisher Scientific, Bremen, Germany) by adding 20 µL BSTFA (*N,O*-Bis(trimethylsilyl)trifluoroacetamide) as a silylation reagent and incubating at 60 °C for 1 h.

A gas chromatograph (TRAC 1310, Thermo Scientific) coupled to a Thermo Fisher Scientific Q Exactive™ GC Orbitrap™ (Dreieich, Hessen, Germany) was used to measure the metabolic profiles. New deactivated glass liners with glass wool were used for each batch of 20 samples. Samples were analyzed in random order. One microliter (1 µL) of each sample was injected at 250 °C into a Split/Splitless (S/SL) injector in split mode with a ratio of 20:1. The separation was performed on a ZB-Semi Volatiles phase column (30 m × 0.25 mm, i.d., 0.25 µm film thickness, Phenomenex, Torrance, CA, USA). The initial oven temperature was maintained at 80 °C for 2 min, and then subsequently increased by 20 °C min^−1^ to 120 °C (hold for 1 min) followed by an increase of 5 °C min^−1^ to 250 °C and to a final temperature of 320 °C by 10 °C min^−1^ (hold for 2 min). Ion source temperature was set at 300 °C. Helium was used as carrier gas with a constant flow of 1.0 mL min^−1^. Mass measurements were carried out in the EI-positive mode and recorded in the range of 50 to 600 *m*/*z* with a resolution of 120,000 *m*/*z*, an (automatic gain control) AGC target of 1 × 10^6^, and a frequency of 5 scans per second. The ionization voltage was set to 70 eV. Instrument performance was monitored by injecting a blank before measurements and every five samples during the analysis. To confirm the metabolites’ identification, authentic standards were analyzed under identical GC–MS conditions followed by matching the mass spectrum and Kovats indices (Supplementary Table S4). Kovats indices were calculated using the unknown metabolite’s retention time and those of the respective alkanes in the retention index mix, as a method previously described [45].

### 4.5. LC–MS Data Adquisition

Before analysis, each sample was redissolved in 50 µl extraction mix solvent (water: methanol: acetonitrile = 1:2:2). LC–MS metabolic profiles were obtained by injecting 1 µL of each sample to an Ultimate 3000 chromatographic system coupled with a Thermo Fisher Scientific Q-Exactive mass spectrometer (Dreieich, Hessen, Germany). Chromatographic separation was obtained on a 100 × 2.1 mm, 2.6 µm reversed-phase Accucore C18 column (Dreieich, Hessen, Germany) and separated with a mobile phase of A: 0.1% formic acid (FA) and 0.2% acetonitrile (ACN) in water, and B: 0.1% FA in ACN as follows: 0–0.2 min isocratic 100% A, 0.2–8 min linear gradient to 100% B, 8–11 min isocratic 100% B, 11.0–12.0 min linear gradient 100% A, 12.1–14 min isocratic 100% A. Samples were, respectively, tested in negative and positive modes by a full scan with acquisition parameters set as follows: a resolution of 70.000, automated gain control (AGC target) of 3 × 10^6^, scan range of 100–1500 *m*/*z*. A pooled QC sample was analyzed using data-dependent MS^2^. The spray voltages were set to 2.5 kV and 3.0 kV for the positive and negative modes, respectively. Full MS/dd-MS^2^ (topN) was based on the full mass spectrum acquired for the top 5 most intense *m*/*z* for both ionization modes to obtain the MS^2^ spectra of each compound with the following settings: Resolution of 17.500, AGC target of 1 × 10^5^, max IT of 50 ms, isolation window of 0.4 *m*/*z*, and normalized collision energy from 15, 30, and 45 eV.

### 4.6. Data Analysis

Raw data files were converted to mzXML format by using the file converter tool MSConvert (ProteoWizard 3.0). Data processing was carried out under R 3.3.3 environment with the XCMS [46] and CAMERA package [47]. For GC data, matchedFilter method was used for the selection of peaks (whm = 3, step = 0.001, steps = 2, max = 50, snthresh = 3, mzdiff = 0.001), following by orbiwarp to correct the retention times of selected peaks and a second alignment with the density method (using bw = 2). For LC data, centWave method was used (ppm = 1, prefilter = c (6, 50,000), peakwidth = c (5, 20), mzCenterFun = “wMean”, BPPARAM = bpparam (), fitgauss = TRUE, integrate = 1, verbose.columns = TRUE, mzdiff = −0.001, sleep = 0). Afterward, peaks were extracted by removing the background ions from negative control samples and features with a relative standard deviation (RSD) of >20% (LC–MS) or >30% (GC–MS) in QC samples over the entire run.

Data scaling and transformation were performed using the MetaboAnalyst 3.0 web-based tool [48]. Principal component analysis (PCA) was executed to obtain an overview of the relationships among the metabolic profiles from all treatments. A partial least squares-discriminant analysis (PLS-DA) was performed to confirm the differences among the metabolic profiles of all treatments. A *t*-test analysis was used to calculate the statistical significance (*p* < 0.05) and fold change of selected metabolites. Differentially modulated metabolites were selected based on their variable importance in projection (VIP) scores, *p*-values, and fold changes. Putative identification for the selected discriminant metabolites of GC–MS data was carried out by searching mass spectra against libraries such as the NIST/EPA/NIH Mass Spectral Library using the MS Search software 2.0 and the Human Metabolome Database. Significantly discriminant metabolites from LC–MS data were putatively identified by the comparison of MS^2^ spectra acquired with those available in the following databases: SIRIUS 4 [49], CFM-ID [50], MetFrag [51], mzCloud [52], and LipidBank [53]. Confidence levels of compound annotations match those from a study previously published. A pathway enrichment analysis was carried out for the differentially modulated metabolites from the GC–MS analysis using MetaboAnalyst 3.0 with the model organism *Arabidopsis thaliana* as a reference. Selected critical pathways were further checked in the *G. sulphuraria* pathway library on The Kyoto Encyclopedia of Genes and Genomes pathway database [54].

## 5. Conclusions

The extremophilic microalgae *G. sulphuraria* has developed metabolic flexibility to overcome stressful environmental conditions. In the current study, a non-targeted UPLC–MS and GC–MS-based metabolomics approach was applied to screen how metabolism in *G. sulphuraria* is modulated in response to high-light intensity exposure and mixotrophic conditions. Significant differences in the level of membrane lipids, such as ergosterol, phosphatidylcholine, and phosphatidylinositol, suggest that *G. sulphuraria* can adjust its lipid rafts to maintain optimal membrane thickness and fluidity under high-light stress. Moreover, *G. sulphuraria* also alters its amino acid pools and accumulates polyamines as a strategy to minimize the potential damage caused by high-light intensity. In mixotrophic *G. sulphuraria,* the accumulation of various osmoprotectants was recorded and interpreted as a strategy to cope with the stress of being exposed to high levels of dissolved organic carbon. To maintain energy homeostasis, mixotrophic *G. sulphuraria* modulates metabolites involved in energy pathways, including the TCA cycle, the shikimate pathway, and the salvage pathway. Our metabolomic findings provide new insights into the metabolic mechanisms used by *G. sulphuraria* cells to handle stressful environmental conditions and open new ways to develop industrial cultures delivering a targeted production of desired compounds.

## Figures and Tables

**Table 1:** Putative identification of top differentially modulated metabolites from *G. sulphuraria* under low-light intensity (L), low-light intensity with glucose addition (L + G), high-light intensity (H), high-light intensity with glucose addition (H + G) culture conditions (LC–MS data).

Comparison	No.	ESI Mode	Putative Compound	Main Class	Formula	Precursor *m*/*z*	RT (min)	PCA Loading (PC1)	VIP	*p*-Value	log2 (FC)	Modulation Trend	Identification Level (Blaženovi´c, Kind, Ji, & Fiehn, 2018)
H vs. L	1	+	LysoPC (18:3)	Lysophospha-tidylcholine	C_26_H_48_NO_7_P	518.3235	6.30	−0.068395	1.043	1.17 × 10^−5^	2.1196	up	Level 1
	2	+	LysoPC (18:2)	Lysophospha-tidylcholine	C_26_H_50_NO_7_P	520.3389	6.65	−0.30333	4.2546	2.5 × 10^−4^	2.5346	up	Level 1
	3	+	LysoPC (18:1)	Glycerophos-phocholine	C_26_H_52_NO_7_P	522.3551	7.44	−0.24913	4.0773	8.18 × 10^−4^	2.3811	up	Level 1
	4	+	PC (18:1/20:6)	Glycerophos-phocholine	C_46_H_79_NO_8_P	804.5494	7.61	−0.29671	3.5255	1.89 × 10^−4^	2.161	up	Level 1
	5	+	unknow	unknow	C_29_H_41_ClN_4_O_8_	631.2508	8.77	0.18704	1.0907	7.55 × 10^−4^	−2.179	down	Level 2
	6	+	unknow	unknow	C_33_H_36_N_6_O_8_	645.265	9.38	0.15828	2.2146	1.0307 × 10^−2^	−1.9636	down	Level 2
	7	+	PI (22:6/22:4)	Glycerophos-phoinositols	C_53_H_83_O_13_P	959.566	10.31	−0.25491	1.1747	2.24 × 10^−4^	−1.2848	down	Level 1
L + G vs. L	1	+	LysoPC (18:3)	Lysophospha-tidylcholine	C_26_H_48_NO_7_P	518.3235	6.30	0.070117	1.1649	8.0053 × 10^−9^	4.3014	up	Level 1
	2	+	PC (18:2/20:6)	Phosphatidyl- choline	C_46_H_77_NO_8_P	802.4861	6.80	0.056888	1.0338	2.07 × 10^−4^	2.2225	up	Level 1
	3	+	DL-tryptophyl-DL- asparagyl-DL-tyrosyl-DL-tyrosine	Unknow Peptide	C_33_H_36_N_6_O_8_	645.2656	9.40	−0.064178	1.131	1.3 × 10^−6^	−5.4394	down	Level 1, Level 1
	4	+	DL-tryptophyl-DL-asparagyl-DL-tyrosyl-DL-phenylalanine	Unknow Peptide	C_33_H_36_N_6_O_7_	629.2705	9.66	−0.070266	1.1715	9.62 × 10^−10^	−7.5637	down	Level 1
	5	+	methyl 3-[(4Z,15Z)-12-acetyl-3,8,13,17-tetramethyl-18-(3-methylperoxypropyl)-6,10,14,20-tetrahydroporphyrin-2-yl]propanoate	Porphyrin Derivatives	C_34_H_40_N_4_O_5_	607.2892	9.78	−0.06728	1.122	2.792 × 10^−6^	−4.2453	down	Level 1
	6	+	PI(22:6/22:4)	Glycerophosphoinositols	C_53_H_83_O_13_P	959.566	10.31	−0.070604	1.0771	4.03 × 10^−5^	−1.7165	down	Level 1
	7	−	LysoPE (18:2)	Lysophosphatidylethanolamine	C_23_H_44_NO_7_P	476.2786	6.47	0.02547	1.1401	6.435 × 10^−3^	−2.2383	down	Level 1
H + G vs. H	1	+	[2-hydroxy-3-[(7~[1])-2-methoxy-12-methyloctadeca-7,17-dien-5-ynoyl]oxypropyl] 2-(trimethylazaniumyl)ethyl phosphate	Lysophosphatidylcholines	C_28_H_50_NO_8_P	560.3346	4.81	−0.077822	1.1378	1.78 × 10^−4^	−1.6521	down	Level 1
	2	+	(2~[2],3~{S})-2-amino-3-[hydroxy-[(2~{R})-2-hydroxy-3-[(~{E})-2-oxononadec-10-enoyl]oxypropoxy]phosphoryl]oxybutanoic acid	Lysophosphatidylcholines	C_26_H_48_NO_10_P	566.3092	4.88	−0.086237	1.2197	2.24 × 10^−6^	−2.5654	down	Level 1
	3	+	4-[[(2~{S},3~{S},5~{R})-5-[6-(diphenylcarbamoyloxy)-2-(2-methylpropanoylamino)purin-9-yl]-3-(tritylamino)oxolan-2-yl]methoxy]-4-oxobutanoic acid	unknown	C_50_H_47_N_7_O_8_	874.3572	4.98	−0.033832	1.186	2.35 × 10^−5^	−2.4192	down	Level 1
	4	+	1,1-dicyclohexyl-3-[5-[[(1-ethylsulfonylpyrrolidin-3-yl)amino]methyl]-1,3-thiazol-2-yl]urea	unknown	C_23_H_39_N_5_O_3_S_2_	498.2573	6.04	−0.14908	1.2502	2.49 × 10^−8^	−4.7897	down	Level 1
	5	+	LysoPC (18:1)	Phosphatidylcholine	C_26_H_52_NO_7_P	522.3551	7.44	−0.018656	1.0573	1.418 × 10^−3^	−2.6821	down	Level 1
	6	+	methyl 3-[(4Z,15Z)-12-acetyl-3,8,13,17-tetramethyl-18-(3-methylperoxypropyl)-6,10,14,20-tetrahydroporphyrin-2-yl]propanoate	Porphyrin Derivatives	C_34_H_40_N_4_O_5_	607.2892	9.78	−0.044436	1.243	1.16 × 10^−7^	−3.1398	down	Level 1
	7	+	PI (22:6/22:4)	Glycerophosphoinositols	C_53_H_83_O_13_P	959.566	10.31	−0.08073	1.1664	6.03 × 10^−5^	−2.0112	down	Level 1
	8	−	LysoPC (18:3)	Lysophosphatidylcholine	C_26_H_48_NO_7_P	562.3165	6.3	0.028007	1.0263	1.0308 × 10^−2^	1.1963	up	Level 1
	9	−	unknown	unknown	unknown	547.277	6.91	−0.10462	1.0438	8.562 × 10^−3^	−1.6929	down	Level 2
	10	−	2-Lysophosphatidylcholine	Glycerophosphates	C_26_H_54_NO_7_P	566.3478	7.68	−0.09984	1.072	6.215 × 10^−3^	−1.8005	down	Level 1
	11	−	Methyl phaeophorbide PubChem CID:136857430	Porphyrin Derivatives	C_36_H_38_N_4_O_5_	605.2775	9.65	−0.070852	1.1764	1.435 × 10^−3^	−3.4996	down	Level 1
	12	−	unknown	unknown	C_48_H_84_N_6_O_10_S	935.5877	10.3	−0.095787	1.2273	5.54 × 10^−4^	−2.0954	down	Level 1
H + G vs. L + G	1	+	LysoPC (18:3)	Lysophosphatidylcholine	C_26_H_48_NO_7_P	518.3235	6.3	0.079268	1.0215	1.1551 × 10^−2^	−1.1442	down	Level 1
	2	+	LysoPC (18:2)	Lysophosphatidylcholine	C_26_H_50_NO_7_P	520.3389	6.65	0.040487	1.2975	7.0637 × 10^−3^	−1.1345	down	Level 1
	3	+	PC (18:2/20:6)	Glycerophosphocholines	C_46_H_77_NO_8_P	802.4861	6.8	0.085526	1.1794	5.162 × 10^−3^	−0.49963	down	Level 1
	4	+	LysoPE (18:1)	Lysophosphatidylethanolamine	C_23_H_46_NO_7_P	480.3072	7.05	0.1797	1.3815	7.45 × 10^−4^	−1.1677	down	Level 1
	5	+	LysoPC (18:1)	Glycerophosphocholines	C_26_H_52_NO_7_P	522.3551	7.44	0.05666	1.2869	8.69 × 10^−4^	−0.85099	down	Level 1
	6	+	PC (18:1/20:6)	Glycerophosphocholines	C_46_H_79_NO_8_P	804.5494	7.61	0.086884	1.2142	1.1944 × 10^−2^	−2.0855	down	Level 1
	7	+	PI (22:6/22:4)	Glycerophosphoinositols	C_53_H_83_O_13_P	959.566	10.31	0.0707	1.4764	1.72 × 10^−3^	−0.76443	down	Level 1
	8	−	LysoPE (18:2)	Lysophosphatidylethanolamine	C_23_H_44_NO_7_P	476.2786	6.47	0.083257	1.054	1.0469 × 10^−2^	−0.79701	down	Level 1
	9	−	LysoPC (18:0)	Lysophosphatidylcholines	C_26_H_54_NO_7_P	566.3478	7.68	0.19846	2.6751	1.0173 × 10^−2^	−1.3039	down	Level 1

Note. ESI = electrospray ionisation; RT = retention time; VIP = variable importance in projection; PCA = principal component analysis; FC = fold change; + positive ionization mode; − negative ionization mode.

## Data Availability

The data that supports the findings of this study are available from the corresponding author upon reasonable request.

## Supplementary Materials

The following are available online at https://www.mdpi.com/1422-0067/22/3/1247/s1.
