# Supplementary material for: Untargeted Metabolomics Unveil Changes in Autotrophic and Mixotrophic Galdieria sulphuraria Exposed to High-Light Intensity
Source: Int J Mol Sci. 2021 Jan 27;22(3):1247. doi: 10.3390/ijms22031247 (PMC7865508; doi:10.3390/ijms22031247)
Supplement: Supplementary file 1 [file ijms-22-01247-s001.pdf]

Figure. S1 Principal component analysis (PCA) score plots of intracellular metabolomic profiles of *G. sulphuraria* collected by GC-MS under two different treatments. a: H vs. L; b: LG vs. L; c: H+G vs H; d: H+G vs. L+G.

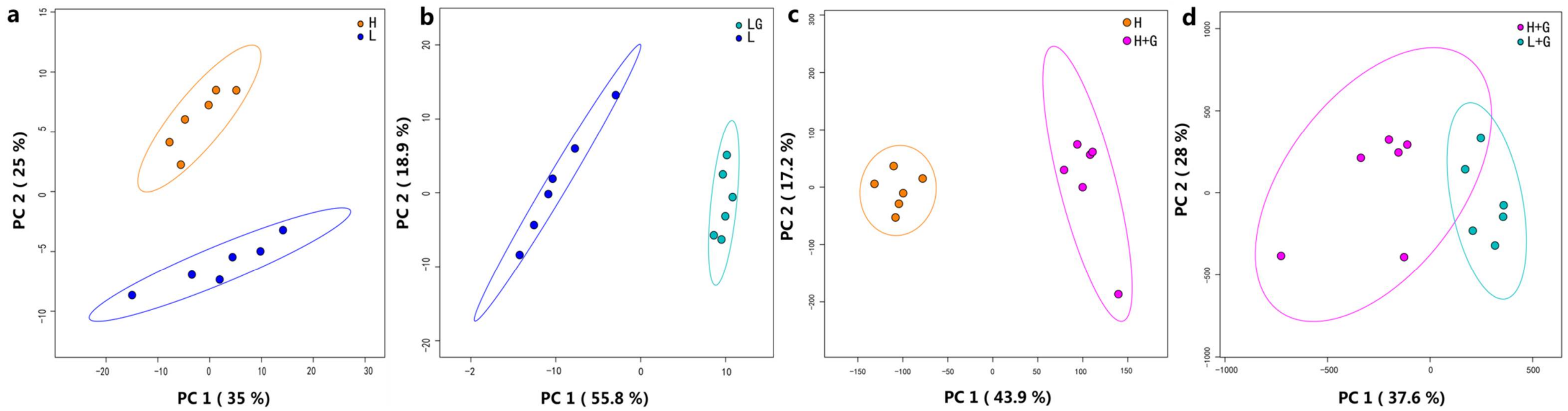

Figure. S2 Principal component analysis (PCA) score plots of intracellular metabolomic profiles of *G. sulphuraria* collected by LC-MS under two different treatments. a: H vs. L; b: LG vs. L; c: H+G vs. H; d: H+G vs. L+G

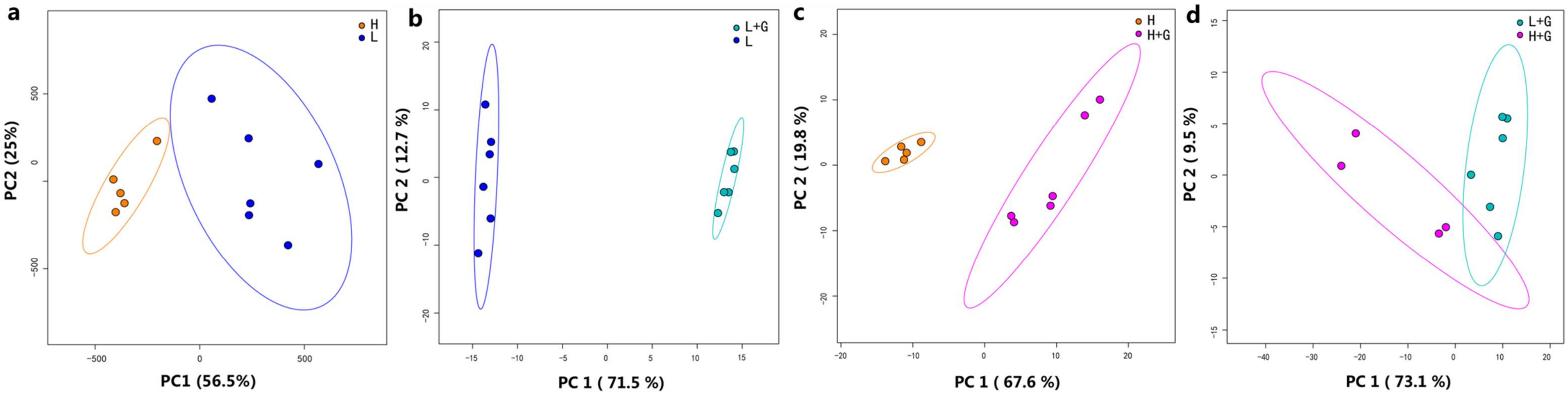

Table S1. Putative identification of top differentially modulated metabolites from *G. sulphuraria* between low-light intensity (L) and high-light intensity (H) culture conditions (GC-MS data)

No.	Putative compound	PCA loading (PC2)	*p* value	VIP	log2(FC)	Base peak	RT(min)	Formula	Modulation trend	Identification level
1	unknow	0.14125	3.78E-08	1.9504	4.5865	275.130109	13.17448	unknow	up	
2	Triquinacene, 1,4,7-tris(methoxy)-	0.14087	1.61E-07	1.9356	3.7918	189.093177	13.2228	$C_{13}H_{16}O_3$	up	Level 2
3	unknow	0.1436	3.62E-07	1.9252	3.2165	205.10739	15.9	$C_{26}H_{36}N_4O_2$	up	
4	Oxymetazoline	0.13188	9.29E-07	1.9107	3.2712	143.052249	14.20431	$C_{16}H_{24}N_2O$	up	Level 2
5	Indole-3-acetamide	0.14619	0.00000132	1.9046	2.608	191.091675	17.038	$C_{10}H_{10}N_2O$	up	Level 2
6	1-Methyl-5-iodouracil	0.13885	0.00000154	1.9017	3.7092	231.103743	15.57347	$C_5H_5IN_2O_2$	up	Level 2
7	Phthalic acid, decyl	0.13374	0.0000127	1.8522	1.9451	149.044797	14.37839	$C_{17}H_{22}O_4$	up	Level 2
8	Hexanoic acid, 2TMS	0.14221	0.0000163	1.8447	2.7875	149.044798	10.50712	$C_{15}H_{34}O_3Si_2$	up	Level 2
9	Propanoic acid, 3TMS	0.13626	0.0000183	1.8411	4.0735	219.12298	13.70793	$C_{13}H_{32}O_4Si_3$	up	Level 2
10	Phytol	-0.1218	0.0000304	1.8243	-1.3011	95.0855102	20.793	$C_{20}H_{40}O$	down	Level 2
11	unknow	0.14037	0.0000306	1.824	3.65	131.052279	13.55474	unknow	up	
12	unknow	0.13307	0.0000445	1.8104	3.4545	131.052297	14.59793	unknow	up	
13	Indole, 3-(4-nitrophenylamino)-	-0.11214	0.0000463	1.8089	-1.6007	253.136637	4.218945	$C_{14}H_{11}N_3O_2$	down	Level 2
14	unknow	0.1373	0.0000717	1.7914	2.0963	230.101338	12.41122	unknow	up	
15	3-Eicosyne	-0.11734	0.000083	1.7851	-1.3392	95.0855092	19.92433	$C_{20}H_{38}$	down	Level 2
16	Phytol, TMS	-0.098629	0.00048073	1.6924	-1.275	143.088618	26.33942	$C_{23}H_{48}OSi$	down	Level 1
17	Ergosterol, TMS	0.020943	0.0014144	1.6145	0.70841	73.0468466	38.57083	$C_{31}H_{52}OSi$	up	Level 1

18	Valine	0.10981	0.0023622	1.5701	1.2635	238.1315	19.32	$C_{11}H_{13}N_3O_6$	up	Level 2
19	unknow	-0.086896	0.003041	1.5462	-1.4893	467.333752	29.35125	unknow	down	
20	unknow	-0.096032	0.0033655	1.5362	-1.2078	273.097301	22.07	unknow	down	
21	unknown	-0.085946	0.0033786	1.5358	-1.3245	409.255762	32.29458	unknown	down	
22	Linoleic acid, methyl ester	-0.09819	0.0035303	1.5314	-1.0164	81.0699249	34.96017	$C_{19}H_{34}O_2$	down	Level 2
23	2-Oxoglutaric acid, 1 MEOX, TMS	0.094883	0.0035547	1.5307	-0.97689	198.057986	14.29172	$C_{12}H_{25}NO_5Si_2$	down	Level 2
24	Oleamide	0.088133	0.0040351	1.5176	-0.9941	72.044401	29.51608	$C_{18}H_{35}NO$	down	Level 2
25	unknow	-0.10108	0.0040717	1.5167	-1.0246	343.019203	9.444142	$C_9H_{27}AsO_4Si_3$	down	Level 2
26	unknow	0.09607	0.0054989	1.4841	1.371	237.111134	12.95065	unknow	up	Level 2
27	Myo-Inositol, 6TMS	-0.085303	0.0068032	1.4595	-1.04438	217.107326	24.69567	$C_{24}H_{60}O_6Si_6$	down	Level 1
28	Leucine, N-ethoxycarbonyl-N-methyl-, tetradecyl ester	0.11174	0.0071779	1.4531	1.4284	172.097114	21.22	$C_{24}H_{47}NO_4$	up	
29	Erythronic acid, 4TMS	-0.010522	0.007428	1.4489	0.74423	129.057248	13.52705	$C_{16}H_{40}O_5Si_4$	up	Level 2
30	Citric acid, 4TMS	0.12718	0.0096879	1.4156	1.0366	273.097239	19.47483	$C_{18}H_{40}O_7Si4$	up	Level 1
31	18-Norabietane	-0.081685	0.012202	1.3848	-0.49204	81.0698905	1.351165	$C_{19}H_{34}$	down	
32	Niacinamide, TMS	0.10443	0.01971	1.3144	0.64915	179.063397	12.17328	$C_9H_{14}N_2OSi$	up	Level 2
33	Putrescine, 4TMS	-0.03746	0.027864	1.2577	3.0134	174.112755	17.6945	$C_{16}H_{44}N_2Si_4$	up	Level 2
34	Glycine, 3TMS	-0.058937	0.04369	1.1753	-1.4528	174.112766	8.564983	$C_{11}H_{29}NO_2Si_3$	down	Level 1
35	Serine, 3TMS	-0.08649	0.096502	1.0004	-2.4475	204.12331	9.5976	$C_{12}H_{31}NO_3Si_3$	down	Level 2

VIP PLS-DA VIP score (variance for Component 1)

Table. S2 Putative identification of top differentially modulated metabolites between mixotrophic and autotrophic *G. sulphuraria* under low-light intensity (L) conditions

NO.	Putative compound	PCA loading (PC1)	*p*-value	VIP	log2(FC)	Base Peak	RT(min)	Formula	Modulation trend	Identification level
1	β-Gentiobiose, octakis(trimethylsilyl) ether	-0.08555	1.46E-08	1.35	-4.64	204.10	27.50	$C_{36}H_{86}O_{11}Si_8$	down	Level 2
2	Glycylglutamic acid, 2TMS	-0.08305	0.0056596	1.02	-1.99	84.04	13.32	$C_{13}H_{27}NO_5Si_2$	down	Level 2
3	Inositol, 6TMS	0.08678	5.18E-08	1.34	2.13	217.11	23.54	$C_{24}H_{60}O_6Si_6$	up	Level 2
4	Glucuronic acid, 5TMS	0.08936	5.82E-09	1.35	4.94	217.11	23.84	$C_{21}H_{50}O_7Si_5$	up	Level 2
5	Citric acid, 4TMS	0.05718	0.005014	1.03	1.13	273.10	19.47	$C_{18}H_{40}O_7Si_4$	up	Level 1
6	Octadecanoic acid, TMS	-0.08227	0.0025139	1.08	-1.12	117.04	27.58	$C_{21}H_{44}O_2Si$	down	Level 1
7	Glycine, 3TMS	-0.09031	0.00031675	1.18	-4.39	174.11	8.56	$C_{11}H_{29}NO_2Si_3$	down	Level 1
8	Uracil, 2TMS	0.08946	1.78E-10	1.36	4.48	241.08	9.17	$C_{10}H_{20}N_2O_2Si_2$	up	Level 2
9	unknow	-0.08331	0.0054126	1.02	-2.12	175.06	8.10	unknow	down	
10	Glucitol, 1,1-di-C-octyl-2,3,4,6-tetra-O-trimethylsilyl-	0.08970	6.01E-06	1.29	3.06	217.11	22.63	$C_{34}H_{78}O_6Si_4$	up	Level 2
11	unknow	0.09038	2.46E-06	1.30	4.26	231.12	23.46	unknow	up	
12	unknow	-0.08371	0.0035319	1.05	-1.98	175.06	8.54	unknow	down	
13	β-Allopyranose, 5TMS	0.08867	1.49E-06	1.31	3.16	217.11	24.81	$C_{21}H_{52}O_6Si_5$	up	Level 2
14	Butanoic acid, 3TMS	0.08468	7.01E-06	1.29	3.64	174.11	13.24	$C_{13}H_{33}NO_2Si_3$	up	Level 2
15	Xylitol, 5TMS	0.08346	0.00035454	1.18	2.25	217.11	17.47	$C_{20}H_{52}O_5Si_5$	up	Level 2
16	Benzene, 1,3,5-tri-tert-butyl-	0.08750	2.80E-07	1.33	3.54	231.09	24.59	$C_{18}H_{30}$	up	Level 2
17	1,4-Butanediamine, 4TMS	0.07594	0.00078428	1.14	2.33	174.11	17.69	$C_{16}H_{44}N_2Si_4$	up	Level 2

18	unknow	0.08993	9.33E-12	1.37	4.19	217.11	17.76	unknow	up	
19	Galactinol, 9TMS	0.08996	6.45E-08	1.34	2.74	204.10	35.08	$C_{39}H_{94}O_{11}Si_9$	up	Level 2
20	Malic acid, 3TMS	0.06927	0.00067057	1.15	1.36	149.04	12.33	$C_{13}H_{30}O_5Si_3$	up	Level 1
21	Threonine, 2TMS	-0.08397	0.003103	1.06	-2.82	130.07	8.33	$C_{10}H_{25}NO_3Si_2$	down	Level 1
22	5-Amino-2-ethyl-1-pentyl-1,2-dihydro-3H-pyrrole-3,3,4-tricarbonitrile	-0.08674	5.07E-06	1.29	-4.46	215.08	16.62	$C_{14}H_{19}N_5$	down	Level 2
23	2-Isopropylmalic acid, 3TMS	0.09007	2.06E-09	1.36	4.50	149.04	14.19	$C_{16}H_{36}O_5Si_3$	up	Level 2
24	Allose, oxime (isomer 1), 6TMS	0.06930	0.0018869	1.09	1.60	319.16	26.48	$C_{24}H_{61}NO_6Si_6$	up	Level 2
25	unknow	-0.09383	3.59E-08	1.34	-3.89	143.09	26.32	$C_{23}H_{48}OSi$	down	
26	unknow	0.08741	3.05E-08	1.34	2.95	255.10	10.44	$C_{18}H_{22}O_2$	up	
27	unknow	0.08739	3.34E-06	1.30	2.34	217.11	22.27	unknow	up	
28	Threitol	0.08460	1.49E-05	1.27	1.48	149.04	12.75	$C_{16}H_{42}O_4Si_4$	up	Level 2
29	7H-Purine, 7-(trimethylsilyl)-2,6-bis[(trimethylsilyl)oxy]-	0.09091	5.00E-13	1.37	6.50	353.13	23.39	$C_{14}H_{28}N_4O_2Si_3$	up	Level 2
30	unknow	-0.09165	0.00035	1.26	-2.81	409.26	32.29	unknow	down	
31	unknow	0.06301	0.0020473	1.09	1.65	102.07	8.50	unknow	up	
32	4-O-Methyl-myo-inositol, 5TMS	0.09035	1.09E-07	1.34	2.70	217.11	22.96	$C_{22}H_{54}O_6Si_5$	up	Level 2
33	unknow	-0.09196	7.73E-05	1.23	-1.36	77.02	8.97	unknow	down	
34	4-Hydroxybenzoic acid, 2TMS	0.08997	1.81E-11	1.37	4.03	267.09	15.40	$C_{13}H_{22}O_3Si_2$	up	Level 2
35	Tyrosine, 3TMS	0.07687	0.00064382	1.15	6.06	218.10	21.85	$C_{18}H_{35}NO_3Si_3$	up	Level 1
36	Glutaric acid, 2TMS	-0.08806	3.56E-06	1.30	-2.23	149.04	19.81	$C_{13}H_{28}O_4Si_2$	down	Level 2
37	8-Hexadecyne	-0.09331	3.29E-07	1.33	-3.50	95.09	19.92	$C_{16}H_{30}$	down	Level 2
38	Levoglucosan, 3TMS	0.08834	1.99E-10	1.36	3.13	217.11	16.98	$C_{15}H_{34}O_5Si_3$	up	Level 2

39	Ethylphosphonic acid, 2TMS	0.06472	0.0019664	1.09	1.93	211.00	10.92	$C_8H_{23}O_4PSi_2$	up	Level 2
40	unknow	-0.09308	2.52E-07	1.33	-4.27	159.09	18.84	unknow	down	
41	Pseudo uridine, 5TMS	0.08997	1.41E-09	1.14	4.50	217.11	29.08	$C_{24}H_{52}N_2O_6Si_5$	up	Level 2
42	unknow	-0.08905	1.82E-05	1.27	-1.67	149.04	20.61	unknow	down	
43	unknow	0.07680	0.00019475	1.20	1.62	217.11	16.47	unknow	up	
44	Phthalic acid, 2-isopropoxyphenyl undecyl ester	0.08644	3.90E-08	1.34	1.98	149.04	15.83	$C_{28}H_{38}O_5$	up	Level 2
45	4H,8H-Benzo[1,2-c:4,5-c'] bis[1,2,5]thiadiazol-4,8-dione	-0.08203	0.0039262	1.05	-2.86	224.08	9.72	$C_6N_4O_2S_2$	down	Level 2
46	1H-Imidazole-4,5-dicarboxylic acid 5-methylamide 4-phenylamide	-0.08978	0.00031412	1.18	-4.81	244.14	10.88	$C_{12}H_{12}N_4O_2$	down	Level 2
47	Hypoxanthine, 2TMS	0.09066	4.27E-12	1.37	5.77	265.09	19.14	$C_{11}H_{20}N_4OSi_2$	up	Level 1
48	Succinylacetone, 2TMS	-0.06865	0.0005283	1.16	-4.93	185.11	17.25	$C_{13}H_{26}O_4Si_2$	down	Level 2
49	Sclareoloxide	-0.09162	4.27E-05	1.25	-1.25	81.07	21.91	$C_{18}H_{30}O$	down	Level 2
50	Shikimic acid, 4TMS	0.08868	1.58E-09	1.36	4.37	204.10	19.36	$C_{19}H_{42}O_5Si_4$	up	Level 2
51	2,5-Diacetyl-terephthalic acid	0.08326	2.57E-05	1.26	3.18	234.98	19.39	$C_{12}H_{10}O_6$	up	Level 2
52	Thiazolo[4,5-d]thiazole, 2,5-diphenyl-	-0.09424	7.85E-08	1.34	-4.02	294.19	19.03	$C_{16}H_{10}N_2S_2$	down	Level 2
53	8-Nitro-1H-quinolin-5-one	-0.09425	9.08E-08	1.34	-5.61	206.15	14.10	$C_9H_6N_2O_4$	down	Level 2
54	2,8,9-Trioxa-5-aza-1-silabicyclo [3.3.3]undecane, TMS	0.07966	1.57E-05	1.27	2.49	174.11	10.54	$C_8H_{17}NO_3Si$	up	Level 2
55	Bicyclo[2.2.2]octanone	-0.09426	8.95E-08	1.34	-4.53	124.08	25.29	$C_8H_{12}O$	down	Level 2
56	unknow	-0.07773	0.0060358	1.01	-1.08	182.09	9.47	unknow	down	

57	unknow	-0.08426	0.0022571	1.08	-1.62	299.07	17.90	unknow	down	
58	2-Methyl-butylamine	0.08718	3.31E-05	1.25	2.70	58.07	31.16	$C_5H_{13}N$	up	Level 2
59	13-Docosenamide, (Z)-	-0.08617	0.00011598	1.22	-1.98	81.07	34.95	$C_{22}H_{43}NO$	down	Level 2
60	Oleamide	-0.09227	1.21E-05	1.28	-2.73	72.04	29.51	$C_{18}H_{35}NO$	down	Level 2
61	Glyceric acid, 3TMS	-0.09261	4.37E-05	1.25	-2.28	189.08	8.99	$C_{12}H_{30}O_4Si_3$	down	Level 2
62	4,4'-Difluoro-2,2'-bis(trifluoromethyl)diphenyl sulfone	0.08449	0.00013535	1.21	2.80	211.00	21.78	$C_{14}H_6F_8O_2S$	up	Level 2
63	Indole, 3-(4-nitrophenylamino)-	-0.09379	3.37E-09	1.35	-6.23	253.14	16.16	$C_{14}H_{11}N_3O_2$	down	Level 2
64	Cycloleucine, N-methoxycarbonyl-, decyl ester	-0.09269	3.56E-05	1.25	-6.22	142.10	18.01	$C_{18}H_{33}NO_4$	down	Level 2
65	7-Octadecyne, 2-methyl-	-0.09346	2.43E-07	1.33	-2.87	81.07	20.42	$C_{19}H_{36}$	down	Level 2
66	Dodecanedioic acid, 2TMS	-0.07843	0.0014246	1.11	-1.30	157.10	15.47	$C_{24}H_{50}O_4Si_2$	down	Level 2
67	Orotic acid, 3TMS	0.07447	2.81E-05	1.26	2.36	254.09	17.93	$C_{14}H_{28}N_2O_4Si_3$	up	Level 1
68	unknow	-0.08976	0.00021608	1.20	-1.01	81.07	22.70	unknow	down	
69	unknow	-0.08023	0.00026336	1.19	-1.72	273.10	22.06	unknow	down	
70	unknow	0.07909	0.001403	1.11	1.13	218.10	14.52	unknow	up	
71	unknow	-0.07221	0.00016408	1.21	-1.87	122.10	31.66	unknow	down	
72	Oleanitrile	-0.08509	2.14E-07	1.33	-2.58	122.10	24.65	$C_{18}H_{33}N$	down	Level 2
73	Fumaric acid, 2TMS	0.08790	4.96E-05	1.24	2.10	72.08	28.27	$C_{18}H_{33}NO_4$	up	Level 1
74	4-Aminobenzoic acid, TMS	0.09088	6.07E-12	1.37	4.98	266.10	19.71	$C_{13}H_{23}NO_2Si_2$	up	Level 2
75	2-Quinolinecarboxylic acid, 2TMS	0.09044	2.2519E-09	1.356	6.7755	231.1073002	24.2767	$C_{16}H_{23}NO_3Si_2$	up	Level 2
76	unknow	0.07345	0.00012542	1.22	1.04	133.07	18.69	unknow	up	
77	dl-2-Aminoadipic acid, 2TMS	0.09016	1.12E-07	1.34	4.62	98.06	15.45	$C_{12}H_{27}NO_4Si_2$	up	Level 2
78	unknow	-0.09128	6.30E-05	1.24	-1.32	191.18	22.36	unknow	down	

79	unknow	-0.09376	2.56E-06	1.30	-2.37	167.06	13.42	unknow	down	
80	Pantothenic acid, 3TMS	0.08994	3.99E-07	1.32	3.15	201.08	22.88	$C_{18}H_{41}NO_5Si_3$	up	Level 1
81	unknow	0.08223	0.00221	1.08	1.45	237.11	12.95	unknow	up	
82	unknow	0.05823	0.0028637	1.07	1.12	231.12	18.06	unknow	up	
83	2-(Methylthio)phenol, trimethylsilyl ether	-0.08047	0.0038104	1.05	-1.15	182.09	9.94	$C_{10}H_{16}OSSi$	down	Level 2
84	3-Iodobenzoic amide	0.08760	4.78E-09	1.35	3.44	247.12	11.95	C_7H_6INO	up	Level 2
85	unknow	0.08772	2.85E-09	1.35	5.86	201.09	11.98	unknow	up	
86	unknow	-0.09166	2.61E-05	1.26	-3.11	467.33	29.34	unknow	down	
87	unknow	0.07856	0.00011266	1.22	1.23	172.10	21.12	unknow	up	
88	unknow	-0.09207	8.58E-06	1.28	-2.87	180.10	12.23	unknow	down	
89	unknow	-0.08340	0.003905	1.05	-1.40	225.05	14.71	unknow	down	
90	unknow	0.08562	1.03E-06	1.31	2.54	146.06	15.55	unknow	up	
91	unknow	-0.09214	2.46E-05	1.26	-1.35	109.10	22.94	unknow	down	
92	Naphthalene, 2,6-bis(1,1-dimethylethyl)-	-0.09383	8.54E-08	1.34	-4.07	89.04	12.41	$C_{18}H_{24}$	down	Level 2
93	unknow	-0.09357	1.94E-08	1.35	-3.21	157.07	17.29	unknow	down	
94	2-Benzylidene-coumaran-3-one	-0.09357	3.14E-08	1.34	-5.55	253.14	16.87	$C_{15}H_{10}O_2$	down	Level 2
95	l-Proline, N-neopentyloxycarbonyl-, heptadecyl ester	0.08678	6.65E-06	1.29	2.16	184.13	17.87	$C_{28}H_{53}NO_4$	up	Level 2
96	unknow	0.08582	5.71E-07	1.32	2.71	170.06	20.45	unknow	up	
97	3-Phenylpropionaldehyde O-pentafluorophenylmethyl-oxime	-0.09143	3.57E-06	1.30	-2.73	181.10	20.00	$C_{16}H_{12}F_5NO$	down	Level 2

98	unknow	-0.09033	0.00034215	1.18	-1.68	207.12	18.72	unknow	down	
99	unknow	-0.09166	6.85E-05	1.23	-1.16	143.05	17.96	unknow	down	
100	unknow	-0.08440	3.10E-05	1.26	-1.72	191.07	12.36	unknow	down	
101	2-Propanone, 1-(2,5-dimethoxyphenyl)-, oxime	-0.09431	1.12E-07	1.34	-4.63	209.14	14.93	$C_{11}H_{15}NO_3$	down	Level 2
102	unknow	-0.08117	0.005691	1.02	-1.33	387.27	26.40	unknow	down	
103	unknow	-0.08731	0.00035444	1.18	-2.47	107.05	23.79	unknow	down	
104	unknow	-0.08799	0.00022365	1.20	-2.31	400.15	23.36	unknow	down	
105	unknow	-0.08024	0.0069325	1.00	-1.28	257.07	11.88	unknow	down	
106	unknow	0.08954	1.16E-11	1.37	4.36	145.08	20.59	unknow	up	
107	unknow	0.08923	7.53E-09	1.35	3.14	117.07	9.93	unknow	up	

VIP PLS-DA VIP score (variance for Component 1)

Table S3 Putative identification of top differentially modulated metabolites between mixotrophic and autotrophic *G. sulphuraria* under high-light (H) intensity condition

NO.	Putative compound	PCA loading (PC1)	*p*-value	Vip	log2(FC)	Base Peak	RT(min)	Formula	Modulation trend	Identification
1	2-Methyl-7-octadecyne	-0.10072	3.6073E-10	1.3608	-2.852	95.08551326	19.91533	$C_{19}H_{36}$	down	Level 2
2	(1H)Quinolin-4-ol-2-one, 8-nitro-	-0.10138	5.1886E-10	1.3599	-5.2323	206.1539289	14.10757	$C_9H_6N_2O_4$	down	Level 2
3	unknown	-0.099119	1.2179E-09	1.3575	-1.9883	81.06986916	20.41308	unknown	down	
4	N-(4-Nitrophenyl)-1H-indol-3-amine	-0.10159	1.2918E-09	1.3573	-6.9762	253.1366802	16.15158	$C_{14}H_{11}N_3O_2$	down	Level 2
5	unknown	-0.099987	1.9514E-09	1.356	-2.3172	81.06987463	20.78383	unknown	down	
6	Bicyclo[2.2.2]octanone	-0.10115	4.3904E-09	1.3531	-3.8872	124.0757634	25.29342	$C_8H_{12}O$	down	
7	unknown	-0.10093	6.6096E-09	1.3514	-4.6929	113.0709594	18.88008	unknown	down	
8	unknown	-0.10117	8.1015E-09	1.3505	-6.5276	189.0931804	13.22464	unknown	down	
9	unknown	0.097035	1.2088E-08	1.3487	4.4855	217.1073324	17.77342	unknown	up	
10	unknown	-0.10123	3.4611E-08	1.3431	-3.1442	84.04437581	13.3301	unknown	down	
11	unknown	-0.1005	3.4247E-08	1.3431	-2.3042	155.0885533	14.93105	unknown	down	
12	Oleamide	-0.1006	4.0673E-08	1.3421	-1.9618	72.04441595	29.51283	$C_{18}H_{35}NO$	down	Level 2
13	β-Glucopyranuronic acid,5TMS	0.097797	4.3132E-08	1.3417	3.4976	217.1072914	23.83775	$C_{21}H_{50}O_7Si_5$	up	Level 2
14	unknown	-0.10029	6.3224E-08	1.3393	-3.1441	294.1882525	19.05817	unknown	down	
15	unknown	0.09689	1.0452E-07	1.3357	2.8498	224.0736814	20.81417	unknown	up	

16	Benzoic acid, 4-hydroxy, diTMS	0.095134	1.1807E-07	1.3348	3.2577	267.0867179	15.4126	$C_{13}H_{22}O_3Si_2$	up	Level 2
17	unknown	0.098055	1.2648E-07	1.3343	3.9951	231.0867279	24.588	unknown	up	
18	unknown	-0.10049	0.000000131	1.334	-4.5773	275.1301367	13.17758	unknown	down	
19	Uracil, 2TMS	0.094835	1.4876E-07	1.333	3.9976	241.0821711	9.177967	$C_{10}H_{20}N_2O_2Si_2$	up	Level 2
20	Iodobenzene	0.095042	1.7324E-07	1.3318	3.333	204.0995224	19.36358	C_6H_5I	up	
21	unknown	-0.09858	1.7568E-07	1.3317	-3.9935	232.1182999	15.90201	unknown	down	
22	unknown	-0.098748	1.9562E-07	1.3308	-3.2072	274.1402035	17.28733	unknown	down	
23	unknown	-0.10044	1.9738E-07	1.3307	-4.7736	230.1013752	12.42452	unknown	down	
24	1-Aminocyclopentanecarboxylic acid, N-methoxycarbonyl-, octyl ester	-0.10128	2.5382E-07	1.3285	-4.8492	142.0975183	18.01625	$C_{26}H_{49}NO_4$	down	Level 2
25	unknown	-0.10026	2.8085E-07	1.3276	-3.6722	180.0950184	12.24796	unknown	down	
26	unknown	-0.10006	3.2165E-07	1.3263	-5.3257	231.1037692	15.57943	unknown	down	
27	Levoglucosan, 3TMS	0.093032	7.5904E-07	1.3176	2.4728	217.107344	16.98683	$C_{15}H_{34}O_5Si_3$	up	Level 2
28	Glyceryl-glycoside, 6TMS	-0.095816	1.1807E-06	1.3124	-7.1797	204.0995287	27.49742	$C_{21}H_{52}O_6Si_5$	down	Level 2
29	18-Norabietane	-0.099294	1.3438E-06	1.3108	-1.1063	81.06991135	21.91358	$C_{19}H_{34}$	down	Level 2
30	unknown	-0.09913	1.5518E-06	1.309	-1.0369	191.1793665	22.36508	unknown	down	
31	unknown	-0.098698	1.6465E-06	1.3082	-2.6448	244.118333	17.01392	unknown	down	
32	unknown	-0.098744	2.6436E-06	1.3017	-5.2574	131.0523092	14.59484	unknown	down	

33	Pantothenic acid，3TMS	0.093146	3.0922E-06	1.2994	2.2922	201.0814716	22.87758	$C_{18}H_{41}NO_5Si_3$	up	Level 1
34	Inositol,6TMS	0.091281	3.6508E-06	1.2969	1.8955	217.1073233	23.5435	$C_{24}H_{60}O_6Si_6$	up	Level 2
35	unknown	-0.099823	4.5079E-06	1.2936	-2.9793	122.0963948	13.41893	unknown	down	
36	Malic acid, 3TMS	0.090489	5.3142E-06	1.2909	3.0599	149.0448012	12.34862	$C_{13}H_{30}O_5Si_3$	up	Level 1
37	3-Sulfanylpropyl diethylborinate	-0.098305	6.3643E-06	1.2878	-3.5967	131.0694382	21.5825	$C_7H_{17}BOS$	down	Level 2
38	unknown	-0.098234	7.3405E-06	1.2853	-1.8354	218.1151546	19.68217	unknown	down	
39	Phenyl p-methoxyphenylethynyl ketone	-0.096942	9.2222E-06	1.2811	-1.7582	159.1167484	9.834658	$C_{16}H_{12}O_2$	down	Level 2
40	2-Isopropylmalic acid,3TMS	0.090761	0.000010197	1.2792	2.5063	149.0448021	14.19263	$C_{16}H_{36}O_5Si_3$	up	Level 2
41	Glucitol, 1,1-di-C-octyl-2,3,4,6-tetra-O-trimethylsilyl-	0.095814	0.000010391	1.2789	2.5097	217.1074495	29.08542	$C_{34}H_{78}O_6Si_4$	up	Level 2
42	unknown	-0.09494	0.000010984	1.2778	-3.0206	149.0447994	10.52583	unknown	down	
43	Glyceric acid-3-phosphate, 4TMS	-0.098222	0.000012219	1.2757	-3.1965	299.0712574	19.23033	$C_{15}H_{39}O_7PSi_4$	down	Level 1
44	Phosphonothioic acid	-0.094336	0.000013156	1.2742	-3.4714	185.1104299	17.25625	$C_{14}H_{14}NO_4PS$	down	Level 2
45	Phthalic acid, butyl 4-nitrophenyl ester	-0.091037	0.000013605	1.2735	-2.7646	149.0448075	14.37364	$C_{18}H_{17}NO_6$	down	Level 2
46	unknown	-0.092935	0.000015124	1.2714	-4.365	219.123094	13.70782	unknown	down	

47	Niacinamide, TMS	-0.096486	0.00001675	1.2692	-2.2622	179.0634026	12.17796	$C_9H_{14}N_2OSi$	down	Level 2
48	unknown	-0.098336	0.000018074	1.2676	-2.6004	89.04168947	24.1735	unknown	down	
49	1,2-Dimethyl-4-tertbutyl-6-cyclopentylbenzene	-0.096595	0.000018488	1.2671	-2.6071	215.0845145	16.61893	$C_{17}H_{26}$	down	Level 2
50	Valine	-0.095542	0.000019319	1.2662	-1.563	238.1315013	19.31917	$C_{11}H_{13}N_3O_6$	down	Level 2
51	unknown	-0.098017	0.000019407	1.2661	-1.3217	154.0794517	12.50999	unknown	down	
52	unknown	0.088797	0.000020681	1.2647	3.7048	234.9765346	19.39775	unknown	up	
53	1-Methoxy-4-[1-(4-methoxyphenyl)-1-methylethyl]-2-methylbenzene	0.08979	0.0000214	1.2639	2.9278	255.0978917	10.44556	$C_{18}H_{22}O_2$	up	Level 2
54	Kynurenic acid,2TMS	0.088517	0.000041241	1.2482	5.7638	231.1072921	24.27792	$C_{16}H_{23}NO_3Si_2$	up	Level 2
55	unknown	-0.096729	0.000042816	1.2472	-4.4637	82.06513833	14.30949	unknown	down	
56	unknown	0.093051	0.000066104	1.2354	2.6912	231.1230112	23.45867	unknown	up	
57	Threitol,4TMS	0.089639	0.000082301	1.2291	1.0664	149.0448019	12.76151	$C_{16}H_{42}O_4Si_4$	up	Level 2
58	unknown	-0.093936	0.000089454	1.2266	-1.4442	98.98419809	12.89142	unknown	down	
59	Phenol, 2-formylamino-4,6-dinitro-	-0.096352	0.000090091	1.2263	-2.5106	199.0784013	9.650675	$C_7H_5N_3O_6$	down	
60	Indole-3-lactic acid, methyl ester	-0.097218	0.00010674	1.2211	-2.0065	130.0683016	8.33755	$C_{12}H_{13}NO_3$	down	
61	unknown	-0.090875	0.00012587	1.2158	-1.1316	344.9763138	11.91393	unknown	down	
62	unknown	0.089047	0.00013794	1.2128	1.9021	217.1073134	22.26783	unknown	up	

63	Phthalic acid, butyl cyclobutyl ester	-0.088918	0.00020118	1.1997	-2.3379	149.0448091	19.81067	$C_{16}H_{20}O_4$	down	Level 2
64	Myo-Inositol, 5TMS	0.085533	0.00020345	1.1993	2.615	217.1073014	22.96425	$C_{21}H_{50}O_6Si_5$	up	Level 2
65	Phthalic acid, butyl cyclobutyl ester	-0.091548	0.00020931	1.1982	-1.4154	149.0448057	20.6	$C_{16}H_{20}O_4$	down	Level 2
66	unknown	-0.094599	0.00022402	1.1958	-1.5102	409.2558544	32.28717	unknown	down	
67	Ergosterol TMS	0.085737	0.00026394	1.1896	1.145	73.04684877	38.56817	$C_{31}H_{52}OSi$	up	Level 1
68	unknown	-0.093046	0.00031511	1.1826	-1.2062	106.0287264	10.39235	unknown	down	
69	Galactinol,9TMS	0.089567	0.00057136	1.1572	2.3574	204.0997297	35.0715	$C_{39}H_{94}O_{11}Si_9$	up	Level 2
70	unknown	-0.089626	0.00057473	1.157	-1.0085	156.0838348	13.04108	unknown	down	
71	4-Aminobenzoic acid, TMS	0.087026	0.00057373	1.157	3.9956	266.1027199	19.71083	$C_{10}H_{15}NO_2Si$	up	Level 2
72	5-Deoxyribofuranose, 3TMS	0.088642	0.00062885	1.1528	1.9859	217.1073045	22.63133	$C_{21}H_{52}O_6Si_5$	up	Level 2
73	Pseudo uridine，5TMS	0.082869	0.0011904	1.1208	3.9514	217.1074495	29.08542	$C_{24}H_{52}N_2O_6Si_5$	up	
74	Xylitol, 5TMS	0.087914	0.0012441	1.1184	1.8836	217.1073495	17.47292	$C_{20}H_{52}O_5Si_5$	up	Level 2
75	Glycine, 3 TMS	-0.090229	0.0015227	1.1072	-1.6748	174.1128493	8.57265	$C_{11}H_{29}NO_2Si_3$	down	Level 1
76	Xanthine, 3TMS	0.080436	0.0019839	1.0916	8.7744	353.1277716	23.38992	$C_{14}H_{28}N_4O_2Si_3$	up	Level 1
77	unknown	-0.086256	0.0028022	1.0698	-1.0615	84.04438462	12.58683	unknown	down	
78	Tyrosine,2TMS	0.073953	0.0029869	1.0655	1.2833	179.0885306	20.80808	$C_{15}H_{27}NO_3Si_2$	up	Level 1

79	unknown	-0.085056	0.0033464	1.0578	-1.022	224.0771308	9.731617	unknown	down	
80	Hypoxanthine, 2TMS	0.077047	0.0034286	1.0562	5.4063	265.0935036	19.14883	$C_{11}H_{20}N_4OSi_2$	up	
81	unknown	-0.083452	0.0034391	1.0559	-2.0736	175.0605011	8.099517	unknown	down	
82	unknown	-0.08555	0.0034479	1.0558	-1.2002	242.0478392	17.96725	unknown	down	
83	Fumaric acid, 2TMS	0.067874	0.003535	1.054	1.112	2.0998	9.42	$C_{18}H_{33}NO_4$	up	Level 1
84	unknown	0.074451	0.0048294	1.0315	3.998	216.0869848	8.505458	unknown	up	
85	unknown	-0.084058	0.0049873	1.0291	-2.0535	215.1209251	25.81392	unknown	down	
86	N-Hydroxynaphthalimide	-0.081714	0.0061691	1.0125	-1.537	183.0947396	14.08312	$C_{10}H_{12}FNO_3$	down	Level 2
87	Phosphoric acid, 3TMS	-0.081575	0.0064227	1.0093	-1.3162	299.0712843	8.039417	$C_9H_{27}O_4PSi_3$	down	Level 2
88	unknown	-0.083058	0.006833	1.0043	-1.4208	298.087479	10.07173	unknown	down	
89	Mannopyranoside, 5TMS	-0.082697	0.0069288	1.0031	-1.8007	204.099702	28.7235	$C_{21}H_{52}O_6Si_5$	down	Level 2

VIP PLS-DA VIP score (variance for Component 1)

Table S4 Putative identification of top differentially modulated metabolites from *G. sulphuraria* between low-light intensity with glucose addition (L+G) and high-light intensity with glucose addition (H+G) culture conditions

No.	Putative compound	PCA loading (PC1)	*p* value	Vip	log2(FC)	Base peak	RT(min)	Formula	Modulation trend	Identification
1	2-O-Glycerol-α-d-galactopyranoside, 6TMS	0.35582	2.3571E-07	5.7399	-3.4786	204.0995	27.49633	$C_{27}H_{66}O_8Si_6$	down	Level 2
2	Ergosterol, TMS	-0.28865	0.00012034	3.9731	1.3541	73.04685	38.56817	$C_{31}H_{52}OSi$	up	Level 1
3	Glutamic acid, 3TMS	0.2788	0.00031649	3.9045	-2.1002	246.1339	15.25761	$C_{14}H_{33}NO_4Si_3$	down	Level 1
4	Malic acid, 3TMS	-0.10462	0.00392	2.2239	1.2202	149.0448	12.348	$C_{13}H_{30}O_5Si_3$	up	Level 1
5	Isopropylmalic acid, 3TMS	0.13929	0.00035804	2.1111	-1.2609	149.0448	14.19663	$C_{16}H_{36}O_5Si_3$	down	Level 2
6	unknown	-0.10078	0.000012739	1.8743	2.946	215.0846	16.62638	unknown	up	
7	15-Hydroxy-7-oxodehydroabietic acid, methyl ester, TMS	-0.15614	0.0027936	1.8621	1.3398	401.0544	6.68424	$C_{24}H_{36}O_4Si$	up	Level 2
8	3,6-Bis(methylsulfanyl)-1,2,4,5-tetraazine	0.076967	0.0017604	1.0175	-1.8912	174.0944	2.901573	$C_4H_6N_4S_2$	down	Level 2

VIP PLS-DA VIP score (variance for Component 1)

Table S5 Compound identifications with pure analytical standards (GC-MS)

Name	RT(min)	Molecular Weight	Kovats indices
Citric acid, 4TMS	19.47	480.851	1814
Myo-Inositol, 6TMS	24.70	613.2426	2081
Octadecanoic acid,TMS	27.58	356.6584	2238
Glycine,3TMS	8.56	291.61	1312
Malic acid,3TMS	12.33	350.6308	1483
Tyrosine,3TMS	21.85	397.7319	1932
Tyrosine,2TMS	20.81	325.5508	1879
Orotic acid, 3TMS	17.93	372.6396	1740
Pantothenic acid,3TMS	22.88	435.779	2016
Fumaric acid, 2TMS	9.42	260.4344	1349
Xanthine, 3TMS	23.39	368.6542	2013
Hypoxanthine, 2TMS	19.16	280.478	1800
Glyceric acid-3-phosphate, 4TMS	19.23	474.7817	1721
Glutamic acid, 3TMS	15.26	363.6726	1616
Ergosterol, TMS	38.57	468.8295	3232
Phytol, TMS	26.32	368.721	2239

Table S6 Pathways significantly affected from the modulated metabolites identified by GC-MS under low-light intensity (L) with glucose addition (L+G) and, high-light intensity (H) with glucose addition (H+G)

Comparison	Pathway	Metabolite	Total	Hits	P value	Impact
H vs L	Cyanoamino acid metabolism	Glycine, Serine	11	2	0.0084135	0
	Methane metabolism	Glycine, Serine	11	2	0.0084135	0.16667
	Citrate cycle (TCA cycle)	Oxoglutaric acid, Citric acid	20	2	0.027103	0.16769
	Glutathione metabolism	Putrescine, Glycine	26	2	0.044258	0.01108
	Aminoacyl-tRNA biosynthesis	Glycine, Serine, Leucine	67	3	0.054205	0.09302
	Glycine, serine and threonine metabolism	Glycine, Serine	30	2	0.057437	0.39716
	Sulfur metabolism	Serine	12	1	0.14797	0
	Sphingolipid metabolism	Serine	13	1	0.15932	0
	Ascorbate and aldarate metabolism	Myoinositol	15	1	0.1816	0
	Propanoate metabolism	Propionic acid	15	1	0.1816	0
	Nitrogen metabolism	Glycine	15	1	0.1816	0
	Glyoxylate and dicarboxylate metabolism	Citric acid	17	1	0.20332	0.10544
	Alanine, aspartate and glutamate metabolism	Oxoglutaric acid	22	1	0.25527	0.09195
	Inositol phosphate metabolism	Myoinositol	24	1	0.27514	0.25131
	Galactose metabolism	Myoinositol	26	1	0.29452	0
	Valine, leucine and isoleucine biosynthesis	Leucine	26	1	0.29452	0.00085
	Cysteine and methionine metabolism	Serine	34	1	0.36725	0
	Valine, leucine and isoleucine degradation	Leucine	34	1	0.36725	0
	Arginine and proline metabolism	Putrescine	38	1	0.40091	0
	Biosynthesis of unsaturated fatty acids	Linoleic acid	42	1	0.43288	0
	Glucosinolate biosynthesis	Leucine	54	1	0.51942	0

LG vs L	Galactose metabolism	Sorbitol, Myoinositol, Galactinol	26	3	0.033531	0.03589
	Arginine and proline metabolism	Putrescine, Proline, Fumaric acid	38	3	0.086626	0.08669
	Tyrosine metabolism	Tyrosine, Fumaric acid	18	2	0.087824	0.27273
	Citrate cycle (TCA cycle)	Citric acid, Fumaric acid	20	2	0.10536	0.12905
	Aminoacyl-tRNA biosynthesis	Threonine, Glycine, Tyrosine, Proline	67	4	0.11269	0
	Phenylalanine, tyrosine and tryptophan biosynthesis	Tyrosine, Shikimic aci	21	2	0.11447	0.09982
	Isoquinoline alkaloid biosynthesis	Tyrosine	6	1	0.15669	0.5
	Glutathione metabolism	Putrescine, Glycine	26	2	0.16263	0.01108
	Glycine, serine and threonine metabolism	Glycine, L-Threonine	30	2	0.20341	0.35453
	Cyanoamino acid metabolism	Glycine	11	1	0.2688	0
	Methane metabolism	Glycine	11	1	0.2688	0
	Pyrimidine metabolism	Orotic acid, Uracil	38	2	0.28755	0.056
	beta-Alanine metabolism	Pantothenic acid	12	1	0.28941	0
	Glycerolipid metabolism	Glyceric acid	13	1	0.30946	0
	Pantothenate and CoA biosynthesis	Pantothenic acid	14	1	0.32896	0.15
	Ascorbate and aldarate metabolism	Myoinositol	15	1	0.34792	0
	Nitrogen metabolism	Glycine	15	1	0.34792	0
	Fructose and mannose metabolism	Sorbitol	16	1	0.36636	0
	Glyoxylate and dicarboxylate metabolism	Citric acid	17	1	0.38429	0.10544
	Alanine, aspartate and glutamate metabolism	Fumaric acid	22	1	0.46681	0.00575
	Ubiquinone and other terpenoid-quinone biosynthesis	Tyrosine	23	1	0.48197	0

	Inositol phosphate metabolism	Myoinositol	24	1	0.49672	0.25131
	Valine, leucine and isoleucine biosynthesis	Threonine	26	1	0.52499	0
	Biosynthesis of unsaturated fatty acids	Stearic acid	42	1	0.70189	0
	Fatty acid biosynthesis	Stearic acid	49	1	0.75732	0
	Purine metabolism	Hypoxanthine	61	1	0.82991	0
HG vs H	Galactose metabolism	Galactinol, D-Mannose, Sorbitol, Myoinositol	26	4	0.0012398	0.03589
	Fructose and mannose metabolism	Sorbitol, D-Mannose	16	2	0.036759	0
	Tyrosine metabolism	Tyrosine, Fumaric acid	18	2	0.045772	0.27273
	Isoquinoline alkaloid biosynthesis	Tyrosine	6	1	0.11116	0.5
	Cyanoamino acid metabolism	Glycine	11	1	0.19463	0
	Methane metabolism	Glycine	11	1	0.19463	0
	beta-Alanine metabolism	Pantothenic acid	12	1	0.2104	0
	Pantothenate and CoA biosynthesis	Pantothenic acid	14	1	0.24105	0.15
	Ascorbate and aldarate metabolism	Myoinositol	15	1	0.25594	0
	Nitrogen metabolism	Glycine	15	1	0.25594	0
	Citrate cycle (TCA cycle)	Fumaric acid	20	1	0.3263	0.03385
	Purine metabolism	Xanthine, Hypoxanthine	61	2	0.33365	0.04869
	Phenylalanine, tyrosine and tryptophan biosynthesis	Tyrosine	21	1	0.33958	0
	Pyruvate metabolism	Isopropylmalic acid	21	1	0.33958	0
	Alanine, aspartate and glutamate metabolism	Fumaric acid	22	1	0.35261	0.00575
	Ubiquinone and other terpenoid-quinone biosynthesis	Tyrosine	23	1	0.36539	0
	Aminoacyl-tRNA biosynthesis	Glycine, Tyrosine	67	2	0.37704	0

	Inositol phosphate metabolism	Myoinositol	24	1	0.37794	0.25131
	Glutathione metabolism	Glycine	26	1	0.40231	0.01108
	Valine, leucine and isoleucine biosynthesis	2-Isopropylmalic acid	26	1	0.40231	0.04831
	Glycine, serine and threonine metabolism	Glycine	30	1	0.44833	0.21756
	Pyrimidine metabolism	Uracil	38	1	0.53039	0
	Arginine and proline metabolism	Fumaric acid	38	1	0.53039	0
	Amino sugar and nucleotide sugar metabolism	D-Mannose	41	1	0.55803	0
HG vs LG	Nitrogen metabolism	Glutamic acid	15	1	0.034559	0
	Butanoate metabolism	Glutamic acid	18	1	0.041374	0
	Alanine, aspartate and glutamatemetabolism	Glutamic acid	22	1	0.05041	0.33333
	Glutathione metabolism	Glutamic acid	26	1	0.05939	0.07756
	Porphyrin and chlorophyll metabolism	Glutamic acid	29	1	0.066087	0
	Arginine and proline metabolism	Glutamic acid	38	1	0.085989	0.14004
	Aminoacyl-tRNA biosynthesis	Glutamic acid	67	1	0.14819	0
